# An examination of how six reasons for valuing nature are endorsed and associated with pro-environmental behavior across 12 countries

**DOI:** 10.1038/s41598-023-34338-x

**Published:** 2023-05-25

**Authors:** Izzy Gainsburg, Sukanya Roy, Julia Lee Cunningham

**Affiliations:** 1 Ross School of Business, University of Michigan, Ann Arbor, MI 48109 USA; 2 John F. Kennedy School of Government, Harvard University, Cambridge, MA 02183 USA

**Keywords:** Psychology, Human behaviour, Environmental social sciences, Psychology and behaviour

## Abstract

Balanced samples from 12 countries (*N* = 12,000) were surveyed about their reasons for valuing nature and pro-environmental behaviors. Results showed that people were least likely to endorse moral-based reasons for valuing nature, as compared to five other reasons (wellbeing benefits, nature’s intrinsic value, health benefits, economic value, identity-based reasons). However, moral- and identity-based reasons (relative to the other four reasons) for valuing nature were the strongest predictors of pro-environmental behavior across three different methods (correlations, linear mixed models, and relative importance analysis) and two pro-environmental behavior categories (consumer behavior and activism). In other words, the reasons for valuing nature most associated with pro-environmental behavior also garnered the weakest support, presenting a potential dilemma for those hoping to leverage values to promote pro-environmental behavior. We also identify a possible mechanism (awareness of one’s environmental impact) to explain why moral- and identity-based reasons for valuing nature best predict behavior. Finally, we examine between-country variability in the endorsement of the six reasons and the reasons’ associations with pro-environmental behaviors, and country-level factors that may explain between-country variability in these outcomes. We discuss these results in the context of broader literature that has focused on an intrinsic vs. instrumental valuation of nature dichotomy.

## Introduction

Across the world, people value nature for various reasons, including the health benefits it provides humans, its economic benefits for their communities, its beauty, and its intrinsic value^1–5^. Importantly, valuing nature motivates behaviors to protect nature and the environment^6^. Because addressing climate change will require pro-environmental behavior from people worldwide, it is important to understand which reasons for valuing nature best predict pro-environmental behavior, and how this differs across cultures. Surprisingly, however, current knowledge about these questions is limited.

Much of the prior research has focused on comparing reasons for valuing nature that are based in the value of nature itself (i.e., its *intrinsic* value) vs. what nature provides for humans (i.e., its *instrumental* value)^7,8^. Recently, however, scholars have called for a broader conceptualization of people’s reasons for valuing nature^9^ and how these reasons vary across cultures^10^. Here, we do this in several ways. First, we examine reasons discretely, instead of lumping them into dichotomous constructs. Second, we integrate research on moral- and identity- based reasons for valuing nature, both of which are types of *relational values* that fall outside the intrinsic-instrumental dichotomy^9^. Finally, we examine how the associations between reasons and pro-environmental behaviors varies across countries and cultures. Together, these contributions improve our understanding of the reasons people choose to engage in environmental behavior and how these processes vary across countries and cultures.

### Intrinsic and instrumental reasons for valuing nature

Prior research has often grouped people’s reasons for valuing nature into intrinsic or instrumental reasons (although others have theorized other categories, such as “egocentric”^2,11–13^). Instrumental reasons focus on the benefits nature provides for humans, such as recreation, human health, and economic value^2,14^. Intrinsic values ascribe value to nature independent of what it provides humans, such as ascribing value to wild animal welfare or an ecosystem’s health. Interestingly, prior work suggests that intrinsic (vs. instrumental) reasons for valuing nature better predict pro-environmental behavior^7,15^. It is unclear, however, whether all instrumental reasons for valuing nature are weaker than intrinsic ones. Thus, we examine three instrumental reasons: economic, health, and wellbeing.

Economic reasons for valuing nature capture people’s valuation of nature’s economic benefits to individuals, communities, and governments (e.g., natural resources for human consumption, eco-tourism). Health-based reasons for valuing nature stem from nature’s role in human health (e.g., clean drinking water, the development of medicine). Finally, people value nature for its wellbeing benefits (e.g., aesthetic beauty, recreation). Importantly, economic, health, and wellbeing reasons for valuing nature are all associated with pro-environmental behaviors^2,4,16^.

### Beyond the intrinsic-instrumental dichotomy: the role of morality and identity

Although the intrinsic-instrumental dichotomy captures many reasons for valuing nature, scholars have recently called for theory to transcend this dichotomy to include *relational* reasons for valuing nature. Chan and colleagues define relational values as “preferences, principles, and virtues about human-nature relationships”^17^ and add that “relational values are not present in things but derivative of relationships and responsibilities to them.”^9^ Thus, we integrate relational values into existing models by drawing on research on moral- and identity- based reasons for valuing nature, both of fall outside the intrinsic-instrumental dichotomy and have relational properties.

Morality-based reasons for valuing nature are connected to beliefs about what is “right and wrong.” Morals are connected to principles, virtues, and responsibilities (i.e., features of relational values), and thus, scholars have conceptualized moral-based reasons for valuing nature as a type of relational value^17^. Religious groups (who may believe nature is a divine creation) and secular groups (e.g., environmental activists) often experience moral motivations to protect nature^18,19^. Meanwhile, identity-based reasons for valuing nature are connected to people’s self-concept (i.e., how people see themselves), which can comprise traits (e.g., seeing oneself as someone who is characteristically interested in nature), preferences (e.g., awareness that one likes nature), and relationships^20^ (e.g., valuing one’s relationship with nature or the role it serves in one’s interpersonal relationships). Because identity-based reasons involve the relationship between something external (i.e., nature) and the self, scholars have conceptualized identity-based reasons as a relational value.

Relevant to the present research, existing work has shown that moral- and identity-based reasons for valuing nature are associated with behavior^21–23^. Still, it is unclear how these reasons compare to intrinsic and instrumental reasons. There are two reasons to believe that morality and identity may be especially powerful motivators of pro-environmental behavior. First, people are more willing to engage in extreme behaviors to address perceived injustices (e.g., speaking out against injustice at a severe cost to oneself) when the social issue is connected to their morals and identities. Second, people are more willing to engage in behaviors with uncertain or distal benefits when the social issue is connected to their morals and identities^24^. This could particularly be relevant to pro-environmental behavior because the relevant goals are often distal and difficult for an individual to address (e.g., solving climate change).

### Psychological mechanisms bridging the reason-behavior relationship

We also explore three mechanisms that might explain why some reasons are stronger associates than others: *awareness of environmental impact*, *self-efficacy*, and *environmental concern*. Awareness of environmental impact is a belief that captures an understanding of how one’s actions affect the natural world; it has been linked to different reasons for valuing nature in the Value-Belief-Norm model of pro-environmental behavior^25^ and theorized as a cognitive component of one’s connectedness to nature (i.e., feelings of oneness with the natural world). Self-efficacy describes people’s belief that their behavior helps achieve their goals. Environmental concern has been conceptualized in many ways^26^, but at its core represents the affective intensity that people feel toward the environment. We chose these three potential mediators because of their distinction from our reasons for valuing nature and from one another, and because each has been found to independently predict pro-environmental behavior^1,27–29^.

Although each of these variables may mediate each reason-behavior relationship, some mediators may be particularly relevant to specific reason-behavior relationships. For instance, identity-based reasons for valuing nature may lead someone to be particularly attuned to their environmental impact, thereby becoming more likely to protect it^30^. As another example, prior research shows that moralization of a social issue is an antecedent to feelings of efficacy, which can in turn foster collective action behaviors^18^. Altogether, examining these mechanisms can help explain why some reasons for valuing nature are most associated with pro-environmental behavior.

### Between-country and cross-cultural variation in reasons for valuing nature

Psychology has historically devoted disproportionate attention to Western, educated, industrialized, rich, and democratic (WEIRD) populations, limiting models of human behavior^31,32^; cross-cultural efforts have also unduly focused on comparing Western (especially the US) and Eastern countries (especially Japan and China)^33^. However, countries and cultures often have different values, including their reasons for valuing nature. For instance, collectivist (vs. individualist) cultures more often emphasize nature’s intrinsic value^34^. Thus, to more comprehensively examine the generalizability and between-country variability in our findings, we analyze data from 12 countries: Australia, Brazil, China, India, Indonesia, Kenya, Mexico, South Africa, South Korea, United Arab Emirates, United Kingdom, and the United States.

In addition, we examine six country-level factors that may underlie between-country variability: environmental performance, pollution, life expectancy, economic prosperity, individualism-collectivism, and preference for hierarchy. Supporting this possibility, prior research has linked these country-level variables to environmental attitudes and behaviors^35–42^. For example, local levels of air pollution stimulate pro-environmental behavior^43^ and more affluent countries have stronger attitude-behavior relationships^44,45^. Thus, it is also plausible that these reasons explain variability in the six reasons for valuing nature. For example, people in less economically prosperous nations may particularly value nature’s economic benefits; people in countries with lower life expectancy may particularly value nature’s health benefits; people in more polluted countries may particularly value nature’s health benefits; and people living in collectivist countries may place particular weight on nature’s intrinsic value (as this orientation can attune people to goals outside the self). These relationships may also work in the opposite direction (e.g., country-level pollution being associated with lower valuation of nature’s health benefits, explaining their tendency to pollute more).

These six variables are also worth exploring for other reasons beyond their connections to environmental values and behaviors. In particular, environmental performance and pollution is related to the present investigation’s interest in environmental protection; individualism-collectivism and preference for hierarchy is a well-established dimensions in cultural psychology on which countries vary^46^; and economic prosperity and life expectancy are widely studied quality-of-life indicators.

### Research overview

We first examine the endorsement of our six reasons for valuing nature (intrinsic, economic, moral, identity, health, wellbeing), between-country variability in the endorsement of these reasons, and country-level variables that explain this variability. Next, we examine which reasons are most associated with two types of pro-environmental behavior (consumer decisions and activism) using three analytical methods (bivariate correlations, linear mixed models, and relative importance analysis). We also examine between-country variability in the reason-behavior relationships and country-level variables that explain this variability. Finally, we test three mediating variables (awareness of environmental impact, self-efficacy, and environmental concern) that may explain why some reasons for valuing nature are most strongly associated with behavior. Through these analyses, we aim to advance our understanding of the value–behavior relationship within environmental psychology and its cross-cultural variability.

## Results

### Models with endorsement of reasons for valuing nature as outcome variable

#### Do people endorse some reasons for valuing nature more than others?

**Figure 1:**
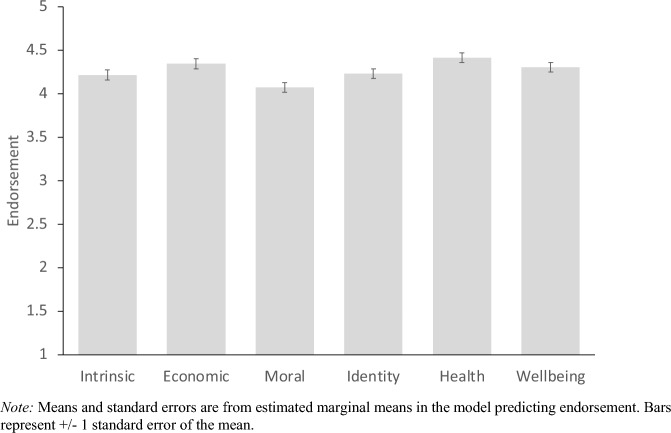
Mean endorsement of each reason for valuing nature.

The first linear mixed model tested whether some reasons for valuing nature were endorsed more than others. The model included “Participant” and “Country” as grouping variables (and modeled a random intercept for Participant and Country). For this model (and all subsequent linear mixed models), the variance of random factors was estimated using the restricted maximum likelihood (REML) method. Endorsement of the six reasons differed significantly from each other *F*(5, 59,995) = 401.0, *p* < 0.001. Figure 1 shows mean agreement for each reason. All pairwise Holm-tests between the different reasons were significant at *p* < 0.001, except the difference between intrinsic and identity-based reasons. Statistics for the model’s random components are reported in Table S1.

#### Does endorsement of different reasons for valuing nature vary across countries?

**Figure 2:**
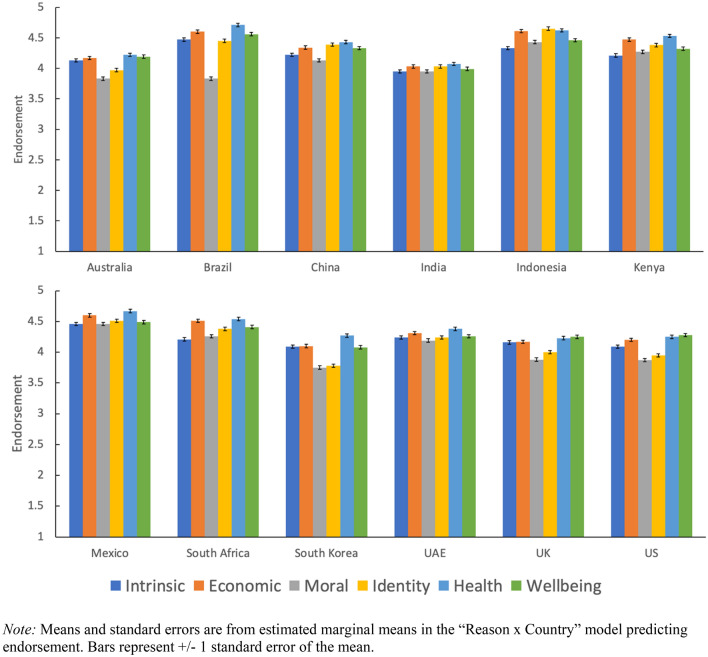
Mean endorsement of each reason for valuing nature across countries.

Next, we examined whether countries varied in their endorsement of reasons for valuing nature by removing Country as a grouping variable and instead entering it as a 12-level fixed factor, along with its interaction with Reason. A significant Reason × Country interaction (*F*(55, 59,940) = 25.9, *p* < 0.001) suggested that countries varied in their endorsement of different reasons. See Fig. 2 for and Table S2 means for each reason in each country and Table S3-S6 for model statistics and simple effects.

Simple effects revealed that the endorsement of different reasons varied most within Brazil (χ^2^ = 1174.5, df = 5, *p* < 0.001), and that the endorsement of different reasons varied least within India (χ^2^ = 26.3, df = 5, *p* < 0.001). Likewise, between-country variability was biggest for the identity reason (χ^2^ = 1174, df = 11, *p* < 0.001) and smallest for the intrinsic reason (χ^2^ = 326, df = 11, *p* < 0.001). As an example of between-country variation, identity-based reasons rated highest among the six reasons in Indonesia, but fifth-highest among the six reasons in the US. There were also similarities across countries: the lowest-rated reason for valuing nature (moral) rated lowest or second-lowest in each country and the highest-rated reason (health) rated highest or second-highest in each country.

Finally, a main effect of Country (*F*(11, 11,988) = 89.7.9, *p* < 0.001) revealed that countries varied in their overall tendency to endorse the different reasons. For instance, overall endorsement was highest in Mexico (*M* = 4.53, 95% CI [4.49, 4.57]) and Indonesia (*M* = 4.53, 95% CI [4.48, 4.56]) and lowest in South Korea (*M* = 4.01, 95% CI [3.97, 4.05]) and India (*M* = 4.00, 95% CI [3.96, 4.04]).

#### What country-level variables explain between-country variability in endorsement of different reasons for valuing nature?

We examined whether six country-level variables (environmental performance, pollution, life expectancy, economic prosperity, individualism-collectivism, and preference for hierarchy) explained between-country variability in endorsement of reasons for valuing nature. We created separate models for each country-level variable, which included the country-level variable (z-scored), Reason, and their interaction. All models included Participant and Country as grouping variables (with random intercepts for Participant and Country) and Endorsement as the dependent variable.

Each country-level variable significantly moderated the effect of Reason on Endorsement. The interaction *F* value was largest for life expectancy and smallest for individualism-collectivism, suggesting that those two variables influenced variability in endorsement among the six reasons the most and least, respectively (see Table S7-S18 for omnibus statistics and random components from all models). Within each model, simple effects revealed the relationship between the country-level variable and the endorsement of each reason (see Table 1). Morality and identity-based reasons were generally the only reasons with country-level predictors: morality and identity-based reasons were endorsed more in countries with lower environmental performance, life expectancy, lower life expectancy, lower economic prosperity, lower individualism, and greater preference for hierarchy. Pollution was the only country-level variable that did not predict endorsement of any reason.

**Table 1:** Simple effects of country-level variables on endorsement of each reason for valuing nature.

Reason	b	SE	Lower	Upper	z	*p*
Country-level variable: environmental performance						
Intrinsic	0.01	0.06	− 0.10	0.13	0.26	.80
Economic	− 0.04	0.06	− 0.15	0.07	− 0.69	.49
Moral	− 0.11	0.06	− 0.22	0.00	− 1.96	.05
Identity	− 0.11	0.06	− 0.22	0.00	− 1.95	.05
Health	− 0.03	0.06	− 0.14	0.08	− 0.51	.61
Wellbeing	0.01	0.06	− 0.10	0.12	0.18	.86
Country-level variable: pollution						
Intrinsic	− 0.06	0.06	− 0.17	0.05	− 1.10	.27
Economic	− 0.07	0.06	− 0.18	0.04	− 1.23	.22
Moral	0.02	0.06	− 0.09	0.13	0.33	.74
Identity	− 0.01	0.06	− 0.12	0.10	− 0.17	.87
Health	− 0.07	0.06	− 0.18	0.04	− 1.27	.20
Wellbeing	− 0.08	0.06	− 0.19	0.03	− 1.44	.15
Country-level variable: life expectancy						
Intrinsic	− 0.02	0.05	− 0.12	0.07	− 0.48	.63
Economic	− 0.11	0.05	− 0.21	− 0.01	− 2.18	.03
Moral	− 0.15	0.05	− 0.25	− 0.05	− 3.02	.003
Identity	− 0.16	0.05	− 0.26	− 0.06	− 3.21	.001
Health	− 0.09	0.05	− 0.19	0.01	− 1.81	.07
Wellbeing	− 0.05	0.05	− 0.15	0.05	− 1.05	.30
Country-level variable: economic prosperity						
Intrinsic	− 0.03	0.05	− 0.14	0.07	− 0.66	.51
Economic	− 0.09	0.05	− 0.20	0.01	− 1.83	.07
Moral	− 0.10	0.05	− 0.20	0.00	− 1.93	.05
Identity	− 0.15	0.05	− 0.25	− 0.05	− 2.96	.003
Health	− 0.09	0.05	− 0.19	0.01	− 1.68	.09
Wellbeing	− 0.05	0.05	− 0.15	0.05	− 0.92	.36
Country-level variable: cultural individualism						
Intrinsic	− 0.05	0.05	− 0.16	0.05	− 1.06	.29
Economic	− 0.08	0.05	− 0.18	0.02	− 1.62	.11
Moral	− 0.12	0.05	− 0.22	− 0.01	− 2.24	.03
Identity	− 0.14	0.05	− 0.24	− 0.03	− 2.62	.01
Health	− 0.10	0.05	− 0.20	0.00	− 1.89	.06
Wellbeing	− 0.03	0.05	− 0.13	0.07	− 0.63	.53
Country-level variable : cultural preference for hierarchy						
Intrinsic	0.05	0.05	− 0.06	0.15	0.92	.36
Economic	0.06	0.05	− 0.04	0.17	1.19	.23
Moral	0.12	0.05	0.02	0.22	2.25	.02
Identity	0.13	0.05	0.03	0.24	2.53	.01
Health	0.07	0.05	− 0.04	0.17	1.24	.21
Wellbeing	0.02	0.05	− 0.08	0.12	0.38	.71

Simple effects of each country-level variable on the endorsement of each reason for valuing nature are derived from separate models, with each model containing the country-level predictor, Reason, and their interaction predicting level of endorsement. The *b* values represent the regression coefficient of the country-level variable predicting endorsement of each reason for valuing nature.

### Models with environmental behavior as outcome variable

#### Which reasons for valuing nature best predict behavior?

Raw correlations between each reason and each pro-environmental behavior (consumer behavior and activism; see Table S19) show the strongest (moral and identity) and weakest (intrinsic) correlates of pro-environmental behavior. These raw correlations are an easily interpretable starting place for examining the reason-behavior relationship, but they do not account for the multilevel structure of our data.

We next ran two linear mixed models (one for each behavior), which account for the multilevel structure of our data by using country as a grouping variable and modeling a random intercept for each country. Because these models include all reasons together (unlike the bivariate correlations), they indicate each reason’s incremental ability to predict behavior when other reasons are held constant. In these two models and all other linear mixed models, we entered the six reasons as z-scored fixed effects (see Table 2 for statistics). For consumer behavior, moral-based (b = 0.12, *p* < 0.001) and identity-based reasons (b = 0.11, *p* < 0.001) best predicted behavior. Health (b = 0.06, *p* < 0.001), economic (b = 0.06, *p* < 0.001), wellbeing (b = 0.06, *p* < 0.001), and intrinsic (b = 0.03, *p* < 0.001) reasons were weaker, but significant, predictors. For activism, the difference between morality- and identity-based reasons and other reasons was larger. Morality-based (b = 0.17, p < 0.001) and identity-based (b = 0.16, *p* < 0.001) were the only positive predictors of behavior; the wellbeing reason was a negative predictor (b =  − 0.03, *p* = 0.04) and the other three reasons were not significant predictors (all *p*s > 0.29).

**Table 2:** Reason–behavior relationships using linear mixed models.

Names	Consumer behavior						Activism behavior					
	b	SE	Lower	Upper	t	*p*	b	SE	Lower	Upper	t	*p*
(Intercept)	3.70	0.06	3.58	3.83	59.04	< .001	2.39	0.09	2.21	2.57	25.94	< .001
Intrinsic	0.03	0.01	0.01	0.04	3.67	< .001	− 0.01	0.01	− 0.03	0.01	− 1.05	0.30
Economic	0.06	0.01	0.04	0.08	6.61	< .001	0.01	0.01	− 0.02	0.03	0.38	0.70
Moral	0.12	0.01	0.10	0.13	13.61	< .001	0.17	0.01	0.14	0.19	13.55	< .001
Identity	0.11	0.01	0.09	0.13	11.85	< .001	0.16	0.01	0.13	0.18	11.65	< .001
Health	0.06	0.01	0.04	0.08	6.21	< .001	0.00	0.01	− 0.02	0.03	0.32	0.75
Wellbeing	0.06	0.01	0.04	0.08	6.40	< .001	− 0.03	0.01	− 0.06	0.00	− 2.06	0.04

The consumer behavior model had an AIC = 24,594 and a Conditional R^2^ = .24. The activism behavior model had an AIC = 33,713 and a Conditional R^2^ = .15. The intraclass correlation coefficient for the random intercept was .09 in both models.

Finally, we conducted two relative importance analyses (one for each outcome; summary results in Table 3) with the six reasons for valuing nature as simultaneous predictors using 12,000 bootstraps to estimate the bias-corrected 95% confidence intervals around the relative weights (e.g., which are akin to adjusted regression coefficients). These models do not account for the multilevel structure of our data, but are most closely tied to our research question concerning which reasons for valuing nature best predict pro-environmental behavior. Relative importance analysis is good at handling intercorrelated predictors (which can cause multicollinearity).

**Table 3:** Relative importance analysis for reasons for valuing nature and pro-environmental behaviors.

Predictor	RW	Lower	Upper	RS-RW (%)
Criterion = Consumer environmental behavior (R2 = .13)				
Intrinsic	0.013	0.010	0.017	9.88
Economic	0.018	0.014	0.022	13.42
Moral	0.035	0.029	0.041	26.08
Identity	0.029	0.024	0.034	21.60
Health	0.020	0.015	0.023	14.15
Wellbeing	0.019	0.016	0.024	14.87
Criterion = Activism environmental behavior (R2 = .08)				
Intrinsic	0.003	0.002	0.004	3.22
Economic	0.007	0.005	0.009	8.03
Moral	0.030	0.025	0.036	37.49
Identity	0.031	0.026	0.037	38.57
Health	0.007	0.005	0.009	8.17
Wellbeing	0.004	0.003	0.005	4.52

RW is the raw relative weights for each reason for valuing nature and can be interpreted as the proportion of variance in behavior that can be attributed to the corresponding reason for valuing nature in the legend. Lower and Upper are the values for the 95% confidence interval for the estimate of the raw relative weight. RS-RW is a “rescaled” relative weight that represents the percentage of the model’s R^2^ that a given reason accounts for.

For consumer behavior, each reason explained significant variance, and their relative importance aligned with the results from the bivariate correlations and linear mixed models: moral-based reasons explained 26.1% of the variance, followed by identity-based reasons for valuing nature (21.6%), wellbeing reasons (14.9%), health reasons (14.1%), economic reasons (13.4%), and nature’s intrinsic value (9.8%). For activism, each reason explained significant variance, and their relative strength aligned with the results of the bivariate correlations and linear mixed models: identity-based reasons explained 38.6% of the variance, followed by moral-based reasons (37.5%), health reasons (8.2%), economic reasons (8.0%), wellbeing reasons (4.5%), and nature’s intrinsic value (3.2%).

#### Do the reason-behavior relationships vary across countries?

We next examined country as a moderator of each reason's capacity to predict behavior. Thus, these models do not use Country as a grouping variable. Overall, these two models suggest that the associations between reasons for valuing nature and pro-environmental behavior vary across countries (see Table 4 for omnibus statistics). For consumer behavior, country moderated the effect of each reason except “health.” For activism, country moderated the effect of all six reasons. When examining these models together, the association between the moral reason and behavior appeared to be most moderated by country—its interaction with country had the second-biggest effect size in the consumer behavior model and the biggest effect size in the activism-behavior model. Table S20-S21 show simple effects of each reason within each country for both dependent variables.

**Table 4:** Country moderation of reason–behavior relationships using linear mixed models.

Predictor	Consumer behavior					Activism behavior				
	SS	df	F	*p*	η^2^p	SS	df	F	*p*	η^2^p
Country	406.74	11	83.30	< .001	0.071	2549.68	11	79.77	< .001	0.069
Intrinsic	5.83	1	13.12	< .001	0.001	827.20	1	0.84	0.36	0.000
Economic	15.40	1	34.70	< .001	0.003	0.79	1	0.23	0.63	0.000
Moral	76.47	1	172.28	< .001	0.014	0.22	1	207.20	< .001	0.017
Identity	55.91	1	125.96	< .001	0.010	195.34	1	90.39	< .001	0.008
Health	12.73	1	28.68	< .001	0.002	85.22	1	0.11	0.75	0.000
Wellbeing	13.18	1	29.70	< .001	0.002	0.10	1	5.64	0.02	0.000
Country × Intrinsic	25.97	11	5.32	< .001	0.005	5.31	11	1.95	0.03	0.002
Country × Economic	9.94	11	2.04	0.02	0.002	20.21	11	1.83	0.04	0.002
Country × Moral	19.82	11	4.06	< .001	0.004	18.94	11	9.84	< .001	0.009
Country × Identity	18.96	11	3.88	< .001	0.004	102.03	11	3.58	< .001	0.003
Country × Health	6.84	11	1.40	0.16	0.001	37.09	11	2.42	0.01	0.002
Country × Wellbeing	11.45	11	2.34	0.01	0.002	25.07	11	1.87	0.04	0.002

The consumer behavior model had an AIC = 24,594 and a Conditional R^2^ = .24. The activism behavior model had an AIC = 33,713 and a Conditional R^2^ = .15. The intraclass correlation coefficient for the random intercept was .09 in both models.

**Figure 3:**
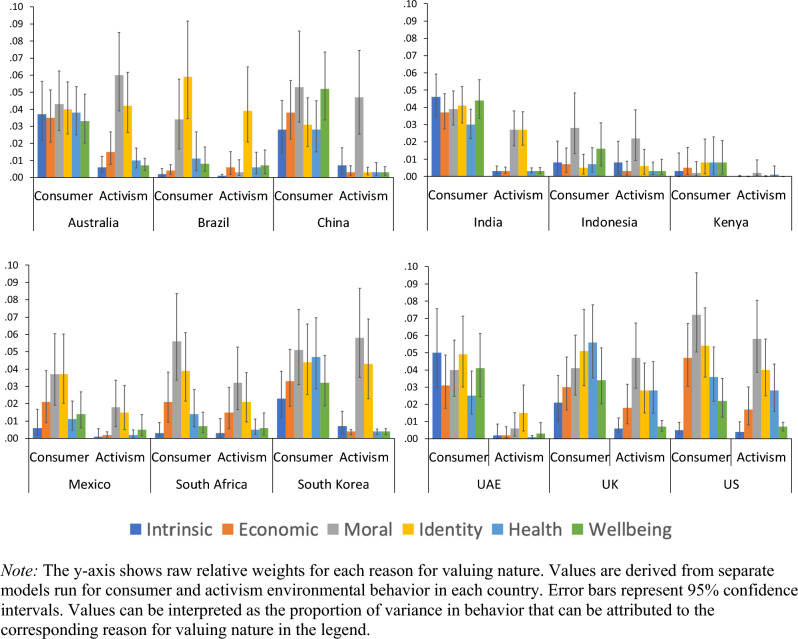
Relative importance of reasons for valuing nature by country and pro-environmental behaviors.

We also ran two relative importance analyses (one for each pro-environmental behavior) within each country and qualitatively examined the rankings of the different reasons across countries (because the data is split by country, country is not used as a variable in these analyses). Figure 3 shows the relative importance of each reason for each outcome in each country (full results are in Table S22).

Results supported the findings from the previous analyses showing that moral- and identity-based reasons were most associated (and intrinsic reasons being last associated) with environmental behavior. For consumer behavior, the moral and identity reasons ranked first or second among in 16 of 24 possible instances. Intrinsic reasons ranked lowest in 6 of 12 possible instances. For activism, moral and identity reasons ranked first or second among in 20 of 24 possible instances; intrinsic reasons ranked lowest in 6 of 12 possible instances.

These analyses also revealed between-country variability. For example, in some countries (e.g., the US, where total R^2^ = 0.24 for consumer behavior), reasons for valuing nature were more associated with pro-environmental behavior than in other countries (e.g., Kenya where total R^2^ = 0.03 for consumer behavior). In addition, the relative importance of the different reasons was not identical across countries. In Indonesia, for instance, identity ranked as the weakest and fourth-weakest predictor of the two measures of behavior, and nature’s intrinsic value ranked as the second- and third-strongest predictors of behavior, deviating from the overall trends. As another example, in India moral reasons (ranked fourth) and identity (ranked third) were relatively middling predictors of consumer behavior, whereas intrinsic reasons best predicted consumer behavior.

#### What country-level variables explain between-country variability in the reason-behavior relationships?

We examined whether the same six country-level variables (environmental performance, pollution, life expectancy, economic prosperity, individualism-collectivism, and preference for hierarchy) moderated the effects of reasons for valuing nature on pro-environmental behavior. We created separate models for each country-level moderator to avoid multicollinearity among country-level predictors and to simplify the models. Each model included Country as a grouping variable and a random intercept for Country.

**Figure 4:**
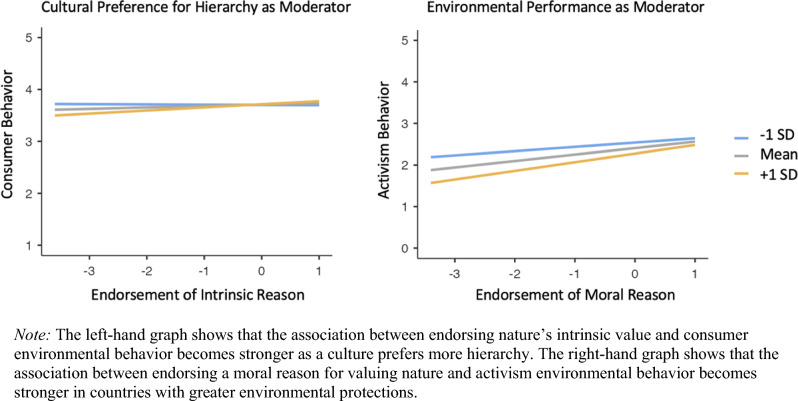
Examples of country-level variables moderating the reason-behavior relationship.

Country-level variables were infrequent moderators in the consumer behavior models (e.g., only 7 of the 36 interactions had *p* values were < 0.05). Cultural preference for hierarchy significantly moderated the effects of three reasons (intrinsic, health, and wellbeing), which was more than any other country-level variable. Country-level variables were significant moderators more often in the activism behavior models (e.g., 27 of the 36 interactions had *p* values < 0.05). Environmental performance moderated the effects of all reasons, which was more than any other country-level variable. We graph two examples of these interactions in Fig. 4 (all main effects for country-level variables and their interactions with reasons are in Table S23).

#### What explains the relative importance of morality- and identity-based reasons for valuing nature in predicting pro-environmental behavior?

We conducted a path analysis to assess whether mediating variables (awareness of environmental impact, self-efficacy, and environmental concern) explained why morality- and identity-based reasons mattered more than other reasons for predicting pro-environmental behavior. We modeled the path analysis using all six reasons for valuing nature, all three mediators, and both measures of behavior. Note that this path analysis does not account for the hierarchical nature of our data (i.e., participants nested within countries). Figure S1 depicts the path analysis model.

**Figure 5:**
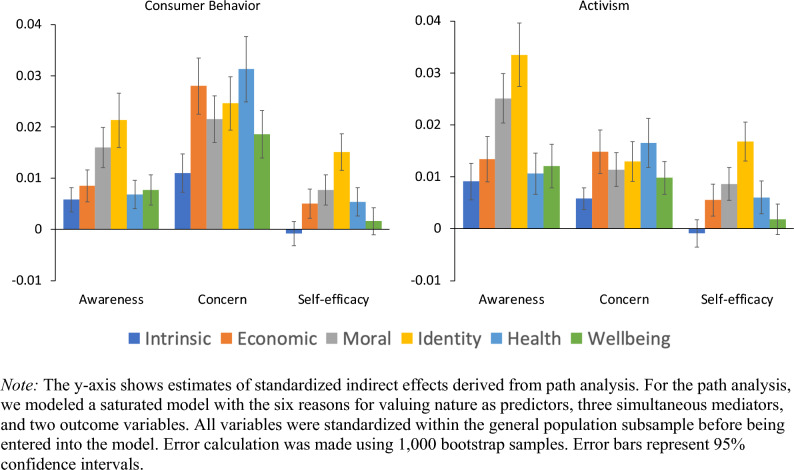
Path analysis indirect effects for mediators of pro-environmental behaviors.

To explore whether any mediators best explained the connection between morality-based and identity-based reasons and pro-environmental behavior, we compared the six indirect effects (one indirect effect for each predictor variable) for each mediator–outcome relationship. If the indirect effects for a given mediator–outcome pathway were significantly greater for identity-based *and* morality-based reasons (vs. the next-highest indirect effect for that pathway), we interpreted this as the mediator particularly relevant to the moral- and identity-based reason-behavior relationships. We determined whether one indirect effect was significantly different from another by specifying the contrast in the model syntax. Indirect effects are graphed in Fig. 5. Parameter estimates are reported in Table S24.

For two of our three mediators (awareness of environmental impact and self-efficacy), indirect effects were strongest for the identity- and morality-based reasons (this true for both pro-environmental behaviors). However, only for awareness of environmental impact were there significant differences between the morality- and identity-based indirect effects and the next-strongest indirect effect (see Fig. 3, where the moral and identity bars on the “awareness” panels are highest). Thus, although the three mediating variables we examined were frequently significant mediators of the reason-behavior relationships, awareness of environmental impact best explains the particularly morality- and identity-based reason-behavior relationships.

## Discussion

The present research compared people’s endorsement of six reasons for valuing nature: its intrinsic value, economic reasons, morality-based reasons, identity-based reasons, health reasons, and wellbeing reasons. We examined which reasons were most endorsed, which best predicted pro-environmental behavior, and how these results varied across 12 countries.

By tapping into the relational values of morality-and identity-based reasons for valuing nature, the present research transcends the intrinsic-instrumental dichotomy. Interestingly, among our six reasons, morality- and identity-based reasons were the strongest predictors of pro-environmental behavior for two measures of behavior (consumer and activism behavior), across several statistical methods, and in the majority of our 12 countries. These findings are interesting considering other research showing the how morality- and identity-based reasons often fail to motivate pro-environmental behavior^47–49^. More research is needed to understand when morality and identity do or do not motivate pro-environmental behavior.

In addition, the relative strength of identity- and morality-based reasons as predictors (vs. other reasons) was more pronounced for activism (vs. consumer) behavior. Although more research is needed to understand why identity- and morality-based reasons were especially important for environmental activism, this finding highlights that research should distinguish between types of pro-environmental behaviors (e.g., Stern et al.’s theory of environmentally significant behavior, which distinguishes between environmental activism, non-activist public sphere behaviors, private sphere environmentalism, and behaviors in organizations)^29^.

Our path analysis identified a possible mechanism (people’s awareness of environmental impact) that helps explain why morality- and identity-based reasons were most associated with pro-environmental behavior. The path analysis showed many showed indirect effects for the three mediators, the six reasons for valuing nature, and the two pro-environmental behaviors; however, the indirect effects of morality- and identity-based reasons were especially strong when awareness of environmental impact was the mediator, highlighting the primary mechanism through which those reasons operated and offering a reminder that different reasons for valuing nature may motivate behavior through different psychological mechanisms. Although more research is needed to understand these processes, one possibility is that holding nature close to one’s identity or moral convictions shifts one’s attention toward their environmental impact.

The present research also reveals an asymmetry: the statistically predictive prominence of moral- and identity-based reasons was observed despite those two reasons being among the least endorsed (moral rated lowest; identity rated third-lowest). Likewise, health-based reasons, the most-endorsed reason, was a relatively weak predictor of pro-environmental behavior. This asymmetry may present a challenge for behavioral change efforts. For instance, messaging campaigns that appeal to the most endorsed reasons (e.g., health) may not best motivate action; likewise, campaigns appealing to the strongest levers (e.g., morality) may not resonate with recipients.

Although our primary findings showed some consistency across countries, we also observed between-country and cross-cultural variability. Endorsement of reasons varied most within Brazil and least within India; likewise, between-country variability was greatest for the identity reason and lowest for the intrinsic reason. The reason-behavior relationships were also moderated by country. The moral reason seemed most moderated by country—its interaction with country had the second-biggest effect size in the consumer behavior model and the biggest effect size in the activism model. Various country-level variables explained the between-country variability in endorsement and in the reason-behavior relationships. One interesting note that dovetails with the broader results in the present research is that moral-based reasons were endorsed more in countries with lower environmental performance indices, but were a stronger predictor of activism in countries with higher environmental performance indices.

This between-country and cross-country variability highlights that environmental behavior research should integrate cross-cultural perspectives into empirical and theoretical work. Our cross-cultural findings are exploratory, and future research could build on these findings to better understand the “why” of the cross-cultural variability. This is difficult because country-level variables are often intercorrelated, because countries typically differ on a multitude of dimensions, and random assignment for country-level predictors is difficult. Hypothesis-driven studies coupled with lab-based manipulations (e.g., priming individualism vs. collectivism) could help unpack the between-country variability in our results.

Finally, we note seven important limitations in the present research. First, leveraging morality- and identity-based reasons for valuing nature in an intervention could backfire if it elicits defensiveness^50,51^ or communicates moral values^50^ and social identities inconsistent with the audience. Moral- and identity-based reasons will likely best motivate behavior when nature and environmentalism are framed in terms of broader, more inclusive moral values or identities^52^ and in ways that are congruent with a recipient’s identities and moral beliefs^53^.

Second, our findings contradict prior work showing that intrinsic (vs. instrumental) reasons are stronger predictors of pro-environmental behavior^7,14,54,55^. Although we confirmed that our measures correlated strongly with longer measures in a separate study and recent advances have validated the use of single-item measures in psychology^56^, our single-item measures may not have adequately captured the underlying constructs. For instance, elements of the items’ wording may explain variance in our outcomes in ways that are disconnected from the overall constructs (e.g., three of our six items explicitly mention protecting or conserving nature). On a more theoretical level, our conclusions may oversimplify the distinction between different reasons for valuing nature. The wellbeing reason, for instance, can fairly be described as relational; likewise, the effects of the moral-based reason may differ if it is connected to people’s perceptions of nature’s intrinsic or instrumental value.

Third, our research only tests three mediating variables, and other mechanisms may better explain the reason-behavior relationships (e.g., goal-setting for pro-environmental behaviors). We were limited by the variables available in the survey and hypothesized mediators after data collection (but prior to testing them). Future research may test other mediators particularly relevant to identity (e.g., self-conscious emotions such as guilt) and morality (e.g., “moral” emotions such as outrage).

Fourth, we cannot infer causality in our research given that it relies on correlational analyses. Future research should explore whether our findings extend to designs that allow for better causal inference, such as laboratory experiments that compare persuasive messages highlighting different reasons for valuing nature. Relatedly, our mediation model cannot “prove” any mechanism due to its correlational nature; experimental designs that manipulate the mediators can address this in future research.

Fifth, because participants took the survey in different languages, it is possible that materials were interpreted differently across countries, which could explain between-country variability. It is also possible that certain constructs manifest differently across countries, requiring scholars to ask different questions for the same constructs across cultures^57,58^.

Sixth, our pro-environmental behavior measure was not validated. Although the items appear straightforward (e.g., the frequency of concrete behaviors during a concrete period, similar to other scales measuring behavioral frequency), it is possible that behaviors have different meanings in different countries or that some behaviors are the default behavior in the local context. In addition, our pro-environmental behaviors do not account for their environmental impact, which limits our understanding of which reasons for valuing nature predict the *most impactful* environmental behaviors (which are often only weakly predicted by attitudinal measures). This is important given the wide variation in the impact of environmental behavior, including some that can have unintentionally negative consequences^59^. Notably, our consumer behaviors are low-impact compared to the most impactful consumer behaviors (e.g., home and vehicle purchases). Future research could address this by extending the present research to validated measures of environmental behavior that weight the relative impact of different environmental behaviors and by using measures that do not rely on self-report (which can be prone to memory and self-presentation biases).

Seventh, our data were collected before the COVID-19 pandemic. It is possible that people’s reasons for valuing nature (and their predictive power) have changed; for instance, due to COVID-19’s hypothesized connection to human-nature interactions, people may now place an increased value on protecting nature for human health.

## Methods

We performed a retrospective analysis of proprietary survey data from the National Geographic Society. All methods were carried out in accordance with the guidelines and regulations of Ipsos (the organization responsible for collecting the data), including the process of obtaining informed consent from participants. We also received approval from the Health Sciences and Behavioral Sciences Institutional Review Board at the University of Michigan to perform the analysis. All survey data is anonymous, did not pose any more than minimal risk to research participants, and only includes participants 18 years and older. For the survey, participants completed a survey consisting of a mix of quantitative, scale-based items and qualitative, open-ended questions.

### Participants

One thousand participants were recruited from 12 different countries (*N* = 12,000) between January 9, 2018 and February 26, 2019 to complete a survey. Quotas were applied to age, gender, and region to make the sample most reflective of the most recent census data in each country. In three of the 10 countries where General Population interviewing was done online (Australia, United Kingdom and United States), internet penetration is sufficiently high to think of the General Population samples as representative of the wider population within the age ranges covered. The remaining online countries (Brazil, China, Indonesia, Mexico, South Africa, South Korea, and the United Arab Emirates) have lower levels of internet penetration, so these samples should not be considered fully nationally representative, and instead represent a more affluent, connected population.

### Procedure

All survey instructions and items were translated into the local language and then back-translated to confirm the accuracy of original translations. Participants first answered screener questions, which collected information about a mixture of demographic variables and other items that would mark them as a “general population” subsample or as eligible for one of the target populations to oversample. These items included their language, age, gender, current region of residence, education, employment sector, income, political behaviors (e.g., contacted a politician), news engagement and interest level, and engagement with National Geographic. These items were tailored to the country as necessary (e.g., income was reported using country-specific currency). Next, they completed the main portion of the survey, which included questions related to valuing nature and pro-environmental behaviors, as well as other questions specific to National Geographic. Surveys were collected online in all countries except in Kenya (face-to-face street intercept) and India (face-to-face door-to-door interviews).

### Materials and measures

The present research focuses on two classes of measures: reasons for valuing nature and pro-environmental behavior. These are listed below, followed by the control variables we used and an explanation of how these were measured.

#### Reasons for valuing nature

Participants indicated their agreement with six items on a 5-point scale (1 = Strongly disagree to 5 = Strongly agree). These six items corresponded to six reasons for valuing nature: Intrinsic, Economic, Moral, Identity, Health, and Wellbeing (see Table 5 for each item). These items were presented in random order. Each item was taken from a separate scale capturing its respective construct. We had to use single-item measures of each scale due to the survey’s space limitations, which are typically adequate proxies for complete scales^35^. We also ran an exploratory factor analysis (see Table S25), which did not reveal any obvious latent variables captured by unique combinations of the six items (e.g., all methods used to choose numbers of factors loaded “intrinsic” onto the same factor as “health” and “wellbeing,” which are theoretically distinct). In addition, all bivariate correlations between the reasons were under 0.60 (see Table S26). These moderate correlations, along with the ambiguous factor structure, lent justification to treating each item as a separate variable.

**Table 5:** Reasons for valuing nature.

Construct	Item	Original scale where item comes from
Intrinsic	Nature has its own value, independent of its value to people	Single-item intrinsic value subscale^9^
Economic	Conserving natural resources is important for the country’s economy	Adapted from single-item economic value subscale^2^
Moral	Conserving nature is a reflection of my core moral beliefs and convictions	Adapted from single-item stewardship/virtue subscale^9^
Identity	Nature is important to me, to who I am as a person	Adapted from single-item individual identity subscale^9^
Health	Protecting nature is important for people’s health	Adapted from single-item health value subscale^2^
Wellbeing	Being in / seeing nature brings people pleasure or satisfaction	Single-item instrumental value subscale^9^

#### Consumer environmental behavior

Participants were asked how frequently they had engaged in four consumer-related environmental behaviors over the past 12 months: “Recycle,” “Avoid products with ingredients that are bad for the environment,” “Use your own reusable shopping bags,” and “Choose to walk, ride a bike, or use public transportation instead of drive.” Frequencies were indicated on a 1 to 5 scale (1 = Never, 2 = Rarely, 3 = Occasionally, 4 = Quite frequently, 5 = All the time). For each participant, we averaged their answers for these four behaviors to create an index of consumer environmental behavior. An exploratory factor analysis offered mixed support for these items loading onto a single factor (all items had factor loadings greater than 0.4, but model fit statistics were relatively weak, e.g., TLI = 0.75 and RMSEA = 0.14; see Table S27 for factor loadings).

#### Activism environmental behavior

Participants were asked whether they had engaged in the following five activism-related environmental behaviors over the past 12 months: “Talk to friends or family about an environmental issue,” “Used social media to share information about an environmental issue,” “Donated money to an environmental cause,” “Volunteered for an environmental cause,” and “Signed a petition to support an environmental cause.” We calculated the sum of these five behaviors as an index of activism environmental behavior. Participants gave answers for “Talk to friends or family about an environmental issue” and “Used social media to share information about an environmental issue” using the (1 = Never to 5 = All the time) scale that was used to measure frequency of consumer environmental behaviors. Because these behaviors had a greater conceptual relationship to activism, rather than the other consumer-related behaviors measured with this question, we dichotomized their answers such that any answer that was a 2 or above was coded as having engaged in the behavior over the past 12 months and a 1 was coded as not having engaged in the behavior in the past 12 months. An exploratory factor analysis offered mixed support for these items loading onto a single factor (all items had factor loadings greater than 0.4, but model fit statistics were only moderate, e.g., TLI = 0.86 and RMSEA = 0.14; see S28 for factor loadings). Although all items describe behaviors that have been conceptualized as activism in prior research, scholars vary as to whether each of these behaviors should be categorized as activism^29,60–63^.

#### Country-level variables in our analysis of country-level moderators

##### Environmental performance index (EPI)

For a country’s environmental performance, we used their measured EPI. When describing EPI. The 2018 EPI report says: “The 2018 Environmental Performance Index (EPI) ranks 180 countries on 24 performance indicators across ten issue categories covering environmental health and ecosystem vitality. These metrics provide a gauge at a national scale of how close countries are to established environmental policy goals. Higher numbers represent better scores. Data were retrieved from the 2018 EPI report^64^.

##### Pollution (PM2.5)

For a country’s level of pollution, we use a measure population-weighted exposure to ambient PM2.5 pollution, which are suspended particles measuring less than 2.5 microns in aerodynamic diameter and capable of penetrating the respiratory tract and causing health damage. Data are from 2017 (the closest measurement date to the time our primary data were collected for the present research) and were retrieved from the World Bank’s website^65^.

##### Life expectancy

This is a population-level measure of the average lifespan of in years within each country. We used life expectancies from 2018 because the data for the present research were also collected in 2018. Data were from the World Bank’s website^66^.

##### Economic prosperity

For a country’s economic prosperity, we used each country’s gross domestic product (GDP) at purchasing power parity (PPP) per capita. We used GDP at PPP (vs. nominal rates in US dollars) because it is typically a better measure of living standards between countries, which we were more interested in given our interest in the behavior of individuals (nominal GDP is more often used to compare national economies on the international market). Data was retrieved from the World Bank’s website^67^.

##### Cultural individualism

For a country’s cultural individualism, we used their score from the “individualism versus collectivism” dimension from Hofstede’s Cultural Dimensions Theory (retrieved from the Hofstede Insights website^68^). When describing this dimension, the Hofstede Insights website writes: “The high side of this dimension, called Individualism, can be defined as a preference for a loosely-knit social framework in which individuals are expected to take care of only themselves and their immediate families. Its opposite, Collectivism, represents a preference for a tightly-knit framework in society in which individuals can expect their relatives or members of a particular ingroup to look after them in exchange for unquestioning loyalty.”

##### Cultural preference for hierarchy

For a country’s cultural preference for hierarchy, we used their score from the “power distance index” from the from Hofstede’s Cultural Dimensions Theory (retrieved from the Hofstede Insights website^68^). When describing this dimension, the Hofstede Insights website writes: “This dimension expresses the degree to which the less powerful members of a society accept and expect that power is distributed unequally. The fundamental issue here is how a society handles inequalities among people. People in societies exhibiting a large degree of Power Distance accept a hierarchical order in which everybody has a place and which needs no further justification. In societies with low Power Distance, people strive to equalize the distribution of power and demand justification for inequalities of power”.

#### Variables used in path analysis

The first mediator we examined in this analysis represented people’s *awareness of environmental impact*, which was a cognitively-oriented item from the connectedness to nature scale^41^ (“I have a deep understanding of how my actions affect the natural world,” 1 = Strongly Disagree, 5 = Strongly Agree). The second mediator represented *self-efficacy*. Participants answered, “Please rate how confident you are that YOU AS AN INDIVIDUAL can attain the following goals in the next 10 years. Please enter a number from 0 to 100 where 0 means ‘cannot do at all,’ 50 means ‘moderately can do’ and 100 means ‘highly certain can do’” for four items: Protect habitats, Reduce plastic pollution in our oceans, Reduce use of fossil fuels (e.g., petroleum, natural gas, coal), Save animals at risk of extinction. The four items were averaged together to create a composite measure of self-efficacy. The third mediator represented *environmental concern*. Participants answered, “How concerned are you personally with each of the following global issues?” (1 = Not at all concerned to 5 = Very concerned) for the following issues: Habitat loss, Plastic pollution, Global climate change, Species at risk of extinction, Air pollution. The four items were averaged together to create a composite measure of environmental concern.

### Data analysis

To examine whether participants endorsed some reasons for valuing nature more than others, we transformed our dataset from “wide” to “long” by creating six rows for each participant (with each row representing a participant’s rating of a single reason for valuing nature). This allowed us to run a linear mixed model with “Reason” as a six-level fixed factor (one level for each reason for valuing nature) and “Endorsement” as a dependent variable (i.e., the endorsement of the reason for valuing nature). We used this “long” dataset for all models with endorsement as the outcome variable. This linear mixed model with the “long” dataset is similar to a repeated measures within-subjects ANOVA with a “wide” dataset and the results lead to the same conclusion^69^.

For all models with environmental behavior as outcome variable, we used a “wide” dataset (i.e., one row for each participant, with separate columns for their responses to all variables).

We note that in our reporting of all regression-based analyses (linear mixed models, linear regression, relative importance analysis, path analysis) that we often use the term “predictors” (i.e., independent variables) and these variables “predicting” outcomes (i.e., dependent variables)—this language strictly reflects a variable’s status in our models as an independent vs. dependent variable and does not imply a causal relationship (which our correlational data cannot support). All data analysis was performed in Jamovi^70^, except for the relative importance analysis (which was performed in RStudio using the R syntax provided from RWA-Web) and the path analysis (which was performed using the SEM module in the JASP statistical program^71^, which uses the *lavaan* package in R).

## Supplementary Information

Supplementary Information.

## Data Availability

The dataset analyzed during the current study is not publicly available due it being proprietary (the data was collected by Ipsos on behalf of the National Geographic Society), but is available from the corresponding author on reasonable request.

## References

[1] Martin L (2020). Nature contact, nature connectedness and associations with health, wellbeing and pro-environmental behaviours. J. Environ. Psychol..

[2] Klain SC, Olmsted P, Chan KMA, Satterfield T (2017). Relational values resonate broadly and differently than intrinsic or instrumental values, or the New Ecological Paradigm. PLoS ONE.

[3] White MP (2019). Spending at least 120 minutes a week in nature is associated with good health and wellbeing. Sci. Rep..

[4] Steinhorst J, Klöckner CA (2018). Effects of monetary versus environmental information framing: Implications for long-term pro-environmental behavior and intrinsic motivation. Environ. Behav..

[5] Schaefer M, Goldman E, Bartuska AM, Sutton-Grier A, Lubchenco J (2015). Nature as capital: Advancing and incorporating ecosystem services in United States federal policies and programs. Proc. Natl. Acad. Sci..

[6] Stern, P. C., Dietz, T., Abel, T. D., Guagnano, G. & Kalof, L. A value-belief-norm theory of support for social movements: The case of environmentalism. *Hum. Ecol. Rev.* **6**, (1999).

[7] Thompson SCG, Barton MA (1994). Ecocentric and anthropocentric attitudes toward the environment. J. Environ. Psychol..

[8] Kortenkamp KV, Moore CF (2001). Ecocentrism and anthropocentrism: Moral reasoning about ecological commons dilemmas. J. Environ. Psychol..

[9] Chan KMA (2016). Opinion: Why protect nature? Rethinking values and the environment. Proc. Natl. Acad. Sci. USA.

[10] Zaval L (2016). Culture and climate action. Nat. Clim. Change.

[11] Stern PC, Dietz T, Guagnano GA (1995). The new ecological paradigm in social-psychological context. Environ. Behav..

[12] Dunlap RE, Van Liere KD, Mertig AG, Jones RE (2000). New trends in measuring environmental attitudes: measuring endorsement of the new ecological paradigm: a revised NEP scale. J. Soc. Issues.

[13] Dunlap RE (2008). The new environmental paradigm scale: From marginality to worldwide use. J. Environ. Educ..

[14] Ford RM, Anderson NM, Nitschke C, Bennett LT, Williams KJH (2017). Psychological values and cues as a basis for developing socially relevant criteria and indicators for forest management. For. Policy Econ..

[15] van der Linden S (2015). Intrinsic motivation and pro-environmental behaviour. Nat. Clim. Change.

[16] Daily, G. C. Management objectives for the protection of ecosystem services. *Environ. Sci. Policy* 333–339 (2000).

[17] Chan KM, Gould RK, Pascual U (2018). Editorial overview: Relational values: What are they, and what’s the fuss about?. Curr. Opin. Environ. Sustain..

[18] van Zomeren M, Postmes T, Spears R (2012). On conviction’s collective consequences: Integrating moral conviction with the social identity model of collective action. Br. J. Soc. Psychol..

[19] van Zomeren M, Pauls IL, Cohen-Chen S (2019). Is hope good for motivating collective action in the context of climate change? Differentiating hope’s emotion- and problem-focused coping functions. Glob. Environ. Change.

[20] Gecas V (1982). The self-concept. Annu. Rev. Sociol..

[21] de Groot JIM, Steg L (2008). Value orientations to explain beliefs related to environmental significant behavior: How to measure egoistic, altruistic, and biospheric value orientations. Environ. Behav..

[22] Schmitt MT, Mackay CML, Droogendyk LM, Payne D (2019). What predicts environmental activism? The roles of identification with nature and politicized environmental identity. J. Environ. Psychol..

[23] Whitmarsh L, O’Neill S (2010). Green identity, green living? The role of pro-environmental self-identity in determining consistency across diverse pro-environmental behaviours. J. Environ. Psychol..

[24] Leary MR, Toner K, Gan M (2011). Self, identity, and reactions to distal threats: The case of environmental behavior. Psychol. Stud..

[25] Hansla A, Gamble A, Juliusson A, Gärling T (2008). The relationships between awareness of consequences, environmental concern, and value orientations. J. Environ. Psychol..

[26] Cruz, S. M. & Manata, B. Measurement of environmental concern: a review and analysis. *Front. Psychol.* **11**, (2020). 10.3389/fpsyg.2020.00363 PMC7067970 32210883

[27] Barbaro N, Pickett SM (2016). Mindfully green: Examining the effect of connectedness to nature on the relationship between mindfulness and engagement in pro-environmental behavior. Personal. Individ. Differ..

[28] Lauren N, Fielding KS, Smith L, Louis WR (2016). You did, so you can and you will: Self-efficacy as a mediator of spillover from easy to more difficult pro-environmental behaviour. J. Environ. Psychol..

[29] Stern PC (2000). New environmental theories: toward a coherent theory of environmentally significant behavior. J. Soc. Issues.

[30] Balundė A, Jovarauskaitė L, Poškus MS (2019). Exploring the relationship between connectedness with nature, environmental identity, and environmental self-identity: A systematic review and meta-analysis. SAGE Open.

[31] Henrich J, Heine SJ, Norenzayan A (2010). Most people are not WEIRD. Nature.

[32] Muthukrishna M (2020). Beyond western, educated, industrial, rich, and democratic (WEIRD) psychology: Measuring and mapping scales of cultural and psychological distance. Psychol. Sci..

[33] Vignoles VL (2016). Beyond the ‘east–west’ dichotomy: global variation in cultural models of selfhood. J. Exp. Psychol. Gen..

[34] Schultz, P. W. Environmental attitudes and behaviors across cultures. *Online Read. Psychol. Cult.* **8**, (2002).

[35] Inglehart R (1995). Public support for environmental protection: Objective problems and subjective values in 43 societies. PS Polit. Sci. Polit..

[36] Mariani F, Pérez-Barahona A, Raffin N (2010). Life expectancy and the environment. J. Econ. Dyn. Control.

[37] Wang Y, Hao F, Liu Y (2021). Pro-environmental behavior in an aging world: Evidence from 31 countries. Int. J. Environ. Res. Public. Health.

[38] Pisano I, Lubell M (2017). Environmental behavior in cross-national perspective: A multilevel analysis of 30 countries. Environ. Behav..

[39] Freymeyer RH, Johnson BE (2010). A cross-cultural investigation of factors influencing environmental actions. Sociol. Spectr..

[40] Duff H, Vignoles VL, Becker M, Milfont TL (2022). Self-construals and environmental values in 55 cultures. J. Environ. Psychol..

[41] Chuang Y, Xie X, Liu C (2016). Interdependent orientations increase pro-environmental preferences when facing self-interest conflicts: The mediating role of self-control. J. Environ. Psychol..

[42] Yang MX, Tang X, Cheung ML, Zhang Y (2021). An institutional perspective on consumers’ environmental awareness and pro-environmental behavioral intention: Evidence from 39 countries. Bus. Strategy Environ..

[43] Sheng G, Dai J, Pan H (2020). Influence of air quality on pro-environmental behavior of Chinese residents: from the perspective of spatial distance. Front. Psychol..

[44] Duroy QMH (2008). Testing the affluence hypothesis: A cross-cultural analysis of the determinants of environmental action. Soc. Sci. J..

[45] Aral ÖH, López-Sintas J (2022). Is pro-environmentalism a privilege? Country development factors as moderators of socio-psychological drivers of pro-environmental behavior. Environ. Sociol..

[46] Hofstede, G. *Culture’s Consequences: International Differences in Work-Related Values*. (SAGE, 1984).

[47] Merritt AC, Effron DA, Monin B (2010). Moral self-licensing: When being good frees us to be bad. Soc. Personal. Psychol. Compass.

[48] Slovic, P., Zionts, D., Woods, A. K., Goodman, R. & Jinks, D. Psychic numbing and mass atrocity. in *The Behavioral Foundations of Public Policy* 126–142 (Princeton University Press, 2013).

[49] Milgram, S. & Gudehus, C. *Obedience to Authority*. (Ziff-Davis Publishing Company, 1978).

[50] Täuber, S. & Zomeren, M. van. Outrage towards whom? Threats to moral group status impede striving to improve via out-group-directed outrage. *Eur. J. Soc. Psychol.* **43**, 149–159 (2013).

[51] Monin B (2007). Holier than me? Threatening social comparison in the moral domain. Rev. Int. Psychol. Soc. Tome.

[52] Kutlaca M, van Zomeren M, Epstude K (2016). Preaching to or beyond the choir: The politicizing effects of fitting value-identity communication in ideologically heterogeneous groups. Soc. Psychol..

[53] Feinberg, M. & Willer, R. Moral reframing: a technique for effective and persuasive communication across political divides. *Soc. Personal. Psychol. Compass* **13**, (2019).

[54] Schultz PW (2005). Values and their relationship to environmental concern and conservation behavior. J. Cross-Cult. Psychol..

[55] Steg L, Dreijerink L, Abrahamse W (2005). Factors influencing the acceptability of energy policies: A test of VBN theory. J. Environ. Psychol..

[56] Matthews RA, Pineault L, Hong Y-H (2022). Normalizing the use of single-item measures: validation of the single-item compendium for organizational psychology. J. Bus. Psychol..

[57] Riordan CM, Vandenberg RJ (1994). A central question in cross-cultural research: Do employees of different cultures interpret work-related measures in an equivalent manner?. J. Manag..

[58] van de Vijver F, Tanzer NK (2004). Bias and equivalence in cross-cultural assessment: an overview. Eur. Rev. Appl. Psychol..

[59] Nielsen K, Cologna V, Lange F, Brick C, Stern P (2021). The case for impact-focused environmental psychology. J. Environ. Psychol..

[60] Poorisat, T., Boster, F. J. & Salmon, C. T. Measures of willingness to engage in activism. (2019).

[61] Seguin C, Pelletier LG, Hunsley J (1998). Toward a model of environmental activism. Environ. Behav..

[62] Lubell M (2002). Environmental activism as collective action. Environ. Behav..

[63] Watson-Singleton NN, Mekawi Y, Wilkins KV, Jatta IF (2021). Racism’s effect on depressive symptoms: examining perseverative cognition and Black Lives Matter activism as moderators. J. Couns. Psychol..

[64] Yale Center for Environmental Law & Policy. *2018 Environmental Performance Index*. https://epi.yale.edu/downloads/epi2018policymakerssummaryv01.pdf (2018).

[65] World Bank. PM2.5 air pollution, mean annual exposure (micrograms per cubic meter). https://data.worldbank.org/indicator/EN.ATM.PM25.MC.M3 (2017).

[66] World Bank. Life expectancy at birth, total (years). https://data.worldbank.org/indicator/SP.DYN.LE00.IN (2018).

[67] World Development Indicators Database, World Bank. GDP per capita, PPP (current international $). https://data.worldbank.org/indicator/NY.GDP.PCAP.PP.CD.

[68] Hofstede Insights. National Culture. https://hi.hofstede-insights.com/national-culture.

[69] Wallace, D. & Green, S. B. Analysis of repeated measures designs with linear mixed models. in *Modeling intraindividual variability with repeated measures data: Methods and applications* 103–134 (Lawrence Erlbaum Associates Publishers, 2002).

[70] The Jamovi Project. Jamovi. (2022).

[71] JASP Team. JASP. (2020).

