# Supplementary material for: An examination of how six reasons for valuing nature are endorsed and associated with pro-environmental behavior across 12 countries
Source: Sci Rep. 2023 May 25;13:8484. doi: 10.1038/s41598-023-34338-x (PMC10209929; doi:10.1038/s41598-023-34338-x)
Supplement: Supplementary file 1 — Supplementary Information. [file 41598_2023_34338_MOESM1_ESM.pdf]

Supplementary Information Appendix (SI) for

An Examination of How Six Reasons for Valuing Nature Are Endorsed and Associated With Pro-environmental Behavior Across 12 Countries

Izzy Gainsburg(*)[a,b], Sukanya Roy[a], & Julia Lee Cunningham[a]

[a] University of Michigan, Ross Schools of Business, Ann Arbor, MI, 48109
[b] Harvard University, John F. Kennedy School of Government, Cambridge, MA, 02183

*Corresponding author: Izzy Gainsburg
E-mail: izzyg@umich.edu

Table S1. Random components from the linear mixed model that examines variation in the endorsement of the six reasons.

Groups	Name	SD	Variance	ICC
Participant	(Intercept)	0.584	0.3409	0.4475
Country	(Intercept)	0.191	0.0365	0.0798
Residual		0.649	0.4209	

Note. Number of Observations: 72000 , groups: Participant 12000, Country 12

Table S2. Raw means and standard deviations of endorsement of reasons by country

	Intrinsic	Economic	Moral	Identity	Health	Wellbeing
Australia	4.13 (0.87)	4.17 (0.87)	3.83 (0.99)	3.97 (0.97)	4.22 (0.88)	4.19 (0.84)
Brazil	4.46 (0.88)	4.60 (0.73)	3.83 (1.10)	4.45 (0.84)	4.71 (0.63)	4.56 (0.71)
China	4.22 (0.75)	4.34 (0.69)	4.13 (0.74)	4.39 (0.71)	4.43 (0.71)	4.33 (0.72)
India	3.95 (1.01)	4.03 (1.05)	3.95 (1.00)	4.03 (0.99)	4.07 (1.03)	3.99 (0.99)
Indonesia	4.33 (0.85)	4.61 (0.67)	4.44 (0.76)	4.65 (0.65)	4.62 (0.67)	4.46 (0.72)
Kenya	4.21 (1.09)	4.47 (0.90)	4.27 (1.00)	4.38 (0.96)		4.32 (1.00)
Mexico	4.46 (0.87)	4.60 (0.70)	4.46 (0.76)	4.51 (0.70)	4.67 (0.64)	4.49 (0.77)
South Africa	4.21 (1.00)	4.51 (0.76)	4.26 (0.85)	4.38 (0.80)	4.54 (0.71)	4.41 (0.77)
South Korea	4.09 (0.85)	4.10 (0.80)	3.75 (0.84)	3.78 (0.83)	4.27 (0.76)	4.08 (0.81)
UAE	4.24 (1.02)	4.31 (0.98)	4.19 (1.03)	4.24 (1.02)	4.38 (0.97)	4.26 (1.00)
UK	4.16 (0.88)	4.17 (0.83)	3.88 (0.91)	4 00 (0.93)	4.23 (0.81)	4.25 (0.82)
US	4.09 (0.95)	4.20 (0.89)	3.87 (1.03)	3.95 (1.00)	4.25 (0.87)	4.28 (0.86)
Total	4.21 (0.93)	4.34 (0.86)	4.07 (0.96)	4.23 (0.91)	4.41 (0.83)	4.30 (0.86)

Note: Means represent levels of agreement with the items on a 5-point scale (1 = Strongly disagree to 5 = Strongly agree).

Table S3. Fixed effect omnibus tests from the linear mixed model that examines variation in the endorsement of the six reasons by country.

	F	Num df	Den df	p
Reason	410.2	5	59940	< .001
Country	89.7	11	11988	< .001
Reason x Country	25.9	55	59940	< .001

Note. Satterthwaite method for degrees of freedom

Table S4. Random components from the linear mixed model that examines variation in the endorsement of the six reasons by country.

Groups	Name	SD	Variance	ICC
Participant	(Intercept)	0.585	0.342	0.454
Residual		0.641	0.411	

Note. Number of Observations: 72000 , groups: Participant 12000, Country 12

Table S5. Simple effects of "Reason" on endorsement within different countries: Omnibus tests

Country	X^2	df	p
US	340.3	5.00	< .001
Mexico	89.3	5.00	< .001
Brazil	1174.5	5.00	< .001
UK	254.8	5.00	< .001
South Africa	208.3	5.00	< .001
Kenya	179.4	5.00	< .001
China	150.4	5.00	< .001
Indonesia	206.1	5.00	< .001
South Korea	504.3	5.00	< .001
India	26.3	5.00	< .001
Australia	286.7	5.00	< .001
UAE	56.8	5.00	< .001

Table S6. Simple effects of "Country" on endorsement of different reasons: Omnibus tests

reason	X^2	df	p
Intrinsic	326	11.0	< .001
Economic	645	11.0	< .001
Moral	913	11.0	< .001
Identity	1092	11.0	< .001
Health	626	11.0	< .001
Wellbeing	406	11.0	< .001

Table S7. Fixed effect omnibus tests from the linear mixed model that examines variation in the endorsement of the six reasons by Environmental Performance

	F	Num df	Den df	p
Reason	403.944	5	59990.0	< .001
Environmental Performance	0.611	1	10.0	0.452
Reason x Environmental Performance	89.172	5	59990.0	< .001

Note. Satterthwaite method for degrees of freedom

Table S8. Random components from the linear mixed model that examines variation in the endorsement of the six reasons by Environmental Performance

Groups	Name	SD	Variance	ICC
Participant	(Intercept)	0.584	0.3414	0.4497
Country	(Intercept)	0.194	0.0378	0.0830
Residual		0.646	0.4178	

Note. Number of Observations: 72000 , groups: Participant 12000, Country 12

Table S9. Fixed effect omnibus tests from the linear mixed model that examines variation in the endorsement of the six reasons by Pollution

	F	Num df	Den df	p
Reason	402.549	5	59990.00	< .001
Pollution	0.666	1	10.00	0.433
Reason x Pollution	47.429	5	59990.00	< .001

Note. Satterthwaite method for degrees of freedom

Table S10. Random components from the linear mixed model that examines variation in the endorsement of the six reasons by Pollution.

Groups	Name	SD	Variance	ICC
Participant	(Intercept)	0.584	0.3411	0.4486
Country	(Intercept)	0.194	0.0376	0.0824
Residual		0.648	0.4193	

Note. Number of Observations: 72000 , groups: Participant 12000, Country 12

Table S11. Fixed effect omnibus tests from the linear mixed model that examines variation in the endorsement of the six reasons by Life Expectancy

	F	Num df	Den df	p
Reason	403.70	5	59990.00	< .001
Life expectancy	3.88	1	10.00	0.077
Reason x Life expectancy	81.97	5	59990.00	< .001

Note. Satterthwaite method for degrees of freedom

Table S12. Random components from the linear mixed model that examines variation in the endorsement of the six reasons by Life Expectancy

Groups	Name	SD	Variance	ICC
Participant	(Intercept)	0.584	0.3413	0.4495
Country	(Intercept)	0.170	0.0288	0.0645
Residual		0.647	0.4181	

Note. Number of Observations: 72000 , groups: Participant 12000, Country 12

Table S13. Fixed effect omnibus tests from the linear mixed model that examines variation in the endorsement of the six reasons by Economic Prosperity

	F	Num df	Den df	p
Reason	402.67	5	59990.0	< .001
Economic Prosperity	2.79	1	10.0	0.126
Reason x Economic Prosperity	51.20	5	59990.0	< .001

Note. Satterthwaite method for degrees of freedom

Table S14. Random components from the linear mixed model that examines variation in the endorsement of the six reasons by Economic Prosperity.

Groups	Name	SD	Variance	ICC
Participant	(Intercept)	0.584	0.3411	0.4485
Country	(Intercept)	0.177	0.0312	0.0692
Residual		0.648	0.4195	

Note. Number of Observations: 72000 , groups: Participant 12000, Country 12

Table S15. Fixed effect omnibus tests from the linear mixed model that examines variation in the endorsement of the six reasons by Cultural Independence

	F	Num df	Den df	p
Reason	402.36	5	59990.0	< .001
Cultural independence	2.84	1	10.00	0.123
Reason x Cultural independence	41.83	5	59990.0	< .001

Note. Satterthwaite method for degrees of freedom

Table S16. Random components from the linear mixed model that examines variation in the endorsement of the six reasons by Cultural Independence.

Groups	Name	SD	Variance	ICC
Participant	(Intercept)	0.584	0.3411	0.4485
Country	(Intercept)	0.177	0.0312	0.0692
Residual		0.648	0.4195	

Note. Number of Observations: 72000 , groups: Participant 12000, Country 12

Table S17. Fixed effect omnibus tests from the linear mixed model that examines variation in the endorsement of the six reasons by Cultural Preference for Hierarchy

	F	Num df	Den df	p
Reason	402.78	5	59990.0	< .001
Cultural Preference for Hierarchy	2.03	1	10.0	0.185
Reason x Cultural Preference for Hierarchy	54.44	5	59990.0	< .001

Note. Satterthwaite method for degrees of freedom

Table S18. Random components from the linear mixed model that examines variation in the endorsement of the six reasons by Cultural Preference for Hierarchy

Groups	Name	SD	Variance	ICC
Participant	(Intercept)	0.584	0.3412	0.4488
Country	(Intercept)	0.183	0.0333	0.0737
Residual		0.647	0.4190	

Note. Number of Observations: 72000 , groups: Participant 12000, Country 12

Table S19. Bivariate Correlations of Reasons for Valuing Nature & Pro-Environmental Behaviors

Predictor	Consumer	Activism
Intrinsic	.22	0.11
Economic	.26	0.16
Moral	.31	0.25
Identity	.30	0.25
Health	.27	0.16
Wellbeing	.27	0.13

Note: All bivariate correlations were statistically significant at the $p < 0.001$ level.

Table S20. Simple effects of different reasons for valuing nature on Consumer Behavior in different countries

Simple effects of Intrinsic reason on Consumer Behavior								
Country	b	SE	Lower	Upper	β	df	t	p
Australia	0.08	0.03	0.03	0.14	0.11	11916	2.88	0.004
Brazil	-0.07	0.03	-0.12	-0.02	-0.09	11916	-2.67	0.01
China	0.05	0.03	-0.02	0.11	0.06	11916	1.46	0.15
India	0.09	0.02	0.05	0.14	0.12	11916	4.30	< .001
Indonesia	0.04	0.03	-0.01	0.09	0.05	11916	1.40	0.16
Kenya	0.02	0.02	-0.02	0.06	0.02	11916	0.90	0.37
Mexico	0.01	0.03	-0.04	0.06	0.01	11916	0.40	0.69
South Africa	-0.01	0.02	-0.05	0.03	-0.01	11916	-0.47	0.64
South Korea	0.03	0.03	-0.03	0.09	0.04	11916	0.90	0.37
UAE	0.12	0.02	0.08	0.17	0.16	11916	5.07	< .001
UK	0.02	0.03	-0.04	0.07	0.02	11916	0.60	0.55
US	-0.05	0.02	-0.10	-0.01	-0.07	11916	-2.26	0.02
Simple effects of Economic reason on Consumer Behavior								
Country	b	SE	Lower	Upper	β	df	t	p
Australia	0.05	0.03	0.00	0.11	0.07	11916	1.89	0.06
Brazil	-0.05	0.03	-0.11	0.01	-0.06	11916	-1.50	0.13
China	0.08	0.03	0.01	0.14	0.10	11916	2.18	0.03
India	0.07	0.02	0.03	0.11	0.09	11916	3.23	0.001
Indonesia	0.01	0.04	-0.06	0.08	0.02	11916	0.39	0.70
Kenya	0.02	0.02	-0.02	0.07	0.03	11916	1.03	0.31
Mexico	0.07	0.03	0.00	0.14	0.09	11916	2.11	0.04
South Africa	0.08	0.03	0.02	0.14	0.10	11916	2.53	0.01
South Korea	0.06	0.03	0.00	0.12	0.08	11916	1.89	0.06
UAE	0.05	0.02	0.00	0.10	0.06	11916	1.96	0.05
UK	0.04	0.03	-0.02	0.09	0.05	11916	1.21	0.23
US	0.13	0.03	0.07	0.18	0.17	11916	4.70	< .001
Simple effects of Moral reason on Consumer Behavior								
Country	b	SE	Lower	Upper	β	df	t	p
Australia	0.08	0.03	0.02	0.14	0.10	11916	2.59	0.01

Brazil	0.10	0.02	0.06	0.14	0.14	11916	5.20	< .001
China	0.13	0.03	0.06	0.19	0.17	11916	3.87	< .001
India	0.09	0.02	0.04	0.13	0.11	11916	3.51	< .001
Indonesia	0.15	0.03	0.09	0.22	0.20	11916	4.46	< .001
Kenya	0.00	0.02	-0.05	0.05	0.00	11916	-0.01	0.99
Mexico	0.14	0.03	0.07	0.21	0.18	11916	4.03	< .001
South Africa	0.21	0.03	0.14	0.27	0.28	11916	6.35	< .001
South Korea	0.12	0.03	0.06	0.18	0.16	11916	3.81	< .001
UAE	0.08	0.03	0.03	0.14	0.11	11916	3.24	0.001
UK	0.06	0.03	0.00	0.12	0.08	11916	1.88	0.06
US	0.19	0.03	0.13	0.24	0.25	11916	6.59	< .001

Simple effects of Identity reason on Consumer Behavior

Country	b	SE	Lower	Upper	β	df	t	p
Australia	0.06	0.03	0.00	0.12	0.08	11916	2.07	0.04
Brazil	0.24	0.03	0.18	0.30	0.32	11916	7.82	< .001
China	0.05	0.04	-0.02	0.12	0.07	11916	1.40	0.16
India	0.08	0.02	0.03	0.12	0.10	11916	3.39	< .001
Indonesia	0.00	0.04	-0.07	0.08	0.00	11916	0.05	0.96
Kenya	0.04	0.02	-0.01	0.09	0.05	11916	1.66	0.10
Mexico	0.15	0.04	0.08	0.22	0.20	11916	4.04	< .001
South Africa	0.14	0.03	0.08	0.21	0.19	11916	4.20	< .001
South Korea	0.11	0.03	0.05	0.16	0.14	11916	3.78	< .001
UAE	0.12	0.03	0.07	0.17	0.16	11916	4.65	< .001
UK	0.09	0.03	0.03	0.15	0.12	11916	2.99	0.003
US	0.12	0.03	0.07	0.18	0.16	11916	4.21	< .001

Simple effects of Health reason on Consumer Behavior

Country	b	SE	Lower	Upper	β	df	t	p
Australia	0.06	0.03	0.00	0.12	0.08	11916	1.97	0.05
Brazil	0.06	0.04	-0.02	0.13	0.07	11916	1.41	0.16
China	0.04	0.03	-0.02	0.10	0.05	11916	1.26	0.21
India	0.04	0.02	0.01	0.08	0.06	11916	2.22	0.03
Indonesia	0.01	0.04	-0.06	0.08	0.02	11916	0.33	0.75
Kenya	0.05	0.02	0.00	0.10	0.06	11916	1.89	0.06

Mexico	0.00	0.04	-0.07	0.07	0.00	11916	0.05	0.96
South Africa	0.01	0.03	-0.05	0.08	0.02	11916	0.40	0.69
South Korea	0.12	0.03	0.05	0.18	0.15	11916	3.63	< .001
UAE	0.01	0.02	-0.04	0.06	0.02	11916	0.50	0.61
UK	0.11	0.03	0.05	0.18	0.15	11916	3.68	< .001
US	0.06	0.03	0.00	0.11	0.08	11916	2.04	0.04

Simple effects of Wellbeing reason on Consumer Behavior								
Country	b	SE	Lower	Upper	β	df	t	p
Australia	0.05	0.03	-0.01	0.11	0.06	11916	1.49	0.14
Brazil	-0.02	0.03	-0.09	0.05	-0.03	11916	-0.60	0.55
China	0.12	0.03	0.05	0.18	0.16	11916	3.57	< .001
India	0.09	0.02	0.05	0.14	0.12	11916	4.29	< .001
Indonesia	0.08	0.03	0.02	0.14	0.11	11916	2.65	0.01
Kenya	0.04	0.02	0.00	0.08	0.05	11916	1.87	0.06
Mexico	0.02	0.03	-0.04	0.08	0.03	11916	0.75	0.46
South Africa	-0.03	0.03	-0.09	0.03	-0.04	11916	-0.96	0.34
South Korea	0.05	0.03	-0.01	0.11	0.06	11916	1.58	0.11
UAE	0.09	0.02	0.04	0.13	0.12	11916	3.69	< .001
UK	0.04	0.03	-0.02	0.11	0.06	11916	1.39	0.16
US	0.02	0.03	-0.04	0.07	0.02	11916	0.57	0.57

Table S21. Simple effects of different reasons for valuing nature on Activism in different countries

Simple effects of Intrinsic reason on Activism								
Country	b	SE	Lower	Upper	β	df	t	p
Australia	-0.01	0.04	-0.09	0.07	-0.01	11916	-0.23	0.82
Brazil	-0.04	0.04	-0.11	0.03	-0.04	11916	-1.11	0.27
China	0.07	0.05	-0.03	0.16	0.06	11916	1.43	0.15
India	0.02	0.03	-0.04	0.08	0.02	11916	0.59	0.56
Indonesia	0.07	0.04	-0.01	0.14	0.06	11916	1.81	0.07
Kenya	0.01	0.03	-0.05	0.06	0.01	11916	0.25	0.80
Mexico	-0.05	0.04	-0.13	0.02	-0.05	11916	-1.43	0.15
South Africa	0.01	0.03	-0.05	0.07	0.01	11916	0.32	0.75

South Korea	-0.15	0.04	-0.24	-0.06	-0.14	11916	-3.39	< .001
UAE	0.01	0.04	-0.06	0.08	0.01	11916	0.40	0.69
UK	-0.03	0.04	-0.12	0.05	-0.03	11916	-0.85	0.40
US	-0.02	0.03	-0.09	0.05	-0.02	11916	-0.60	0.55

Simple effects of Economic reason on Activism

Country	b	SE	Lower	Upper	β	df	t	p
Australia	0.04	0.04	-0.04	0.13	0.04	11916	1.02	0.31
Brazil	0.03	0.05	-0.06	0.12	0.03	11916	0.61	0.54
China	-0.01	0.05	-0.11	0.09	-0.01	11916	-0.27	0.79
India	-0.06	0.03	-0.12	0.00	-0.06	11916	-2.03	0.04
Indonesia	0.00	0.05	-0.11	0.10	0.00	11916	-0.06	0.95
Kenya	-0.01	0.03	-0.08	0.06	-0.01	11916	-0.35	0.73
Mexico	-0.09	0.05	-0.18	0.01	-0.08	11916	-1.82	0.07
South Africa	0.10	0.04	0.01	0.19	0.10	11916	2.28	0.02
South Korea	-0.05	0.04	-0.14	0.04	-0.05	11916	-1.10	0.27
UAE	0.00	0.04	-0.07	0.07	0.00	11916	0.04	0.97
UK	0.08	0.04	-0.01	0.16	0.07	11916	1.82	0.07
US	0.05	0.04	-0.03	0.12	0.04	11916	1.16	0.25

Simple effects of Moral reason on Activism

Country	b	SE	Lower	Upper	β	df	t	p
Australia	0.32	0.04	0.23	0.40	0.29	11916	7.17	< .001
Brazil	0.00	0.03	-0.06	0.06	0.00	11916	-0.05	0.96
China	0.31	0.05	0.22	0.41	0.29	11916	6.52	< .001
India	0.04	0.04	-0.03	0.11	0.04	11916	1.08	0.28
Indonesia	0.20	0.05	0.11	0.30	0.19	11916	4.08	< .001
Kenya	0.04	0.04	-0.03	0.11	0.04	11916	1.14	0.25
Mexico	0.17	0.05	0.07	0.27	0.16	11916	3.41	< .001
South Africa	0.20	0.05	0.11	0.30	0.19	11916	4.22	< .001
South Korea	0.33	0.05	0.24	0.42	0.31	11916	7.13	< .001
UAE	0.04	0.04	-0.03	0.12	0.04	11916	1.11	0.27
UK	0.24	0.05	0.15	0.34	0.23	11916	5.18	< .001
US	0.26	0.04	0.18	0.34	0.24	11916	6.24	< .001

Simple effects of Identity reason on Activism

Country	b	SE	Lower	Upper	β	df	t	p
Australia	0.21	0.04	0.12	0.30	0.19	11916	4.67	< .001
Brazil	0.25	0.04	0.16	0.34	0.24	11916	5.61	< .001
China	-0.01	0.05	-0.11	0.09	-0.01	11916	-0.22	0.83
India	0.06	0.03	0.00	0.13	0.06	11916	1.94	0.05
Indonesia	0.07	0.06	-0.04	0.18	0.06	11916	1.19	0.23
Kenya	0.01	0.04	-0.06	0.08	0.01	11916	0.27	0.79
Mexico	0.16	0.05	0.05	0.26	0.15	11916	2.97	0.00
South Africa	0.12	0.05	0.03	0.22	0.12	11916	2.53	0.01
South Korea	0.21	0.04	0.13	0.29	0.20	11916	5.16	< .001
UAE	0.12	0.04	0.05	0.19	0.11	11916	3.32	< .001
UK	0.12	0.05	0.03	0.21	0.11	11916	2.56	0.01
US	0.16	0.04	0.08	0.25	0.15	11916	3.83	< .001

Simple effects of Health reason on Activism

Country	b	SE	Lower	Upper	β	df	t	p
Australia	-0.03	0.04	-0.11	0.06	-0.03	11916	-0.63	0.53
Brazil	0.01	0.06	-0.10	0.12	0.01	11916	0.17	0.87
China	0.02	0.05	-0.07	0.12	0.02	11916	0.51	0.61
India	-0.04	0.03	-0.10	0.01	-0.04	11916	-1.46	0.14
Indonesia	-0.01	0.05	-0.12	0.09	-0.01	11916	-0.28	0.78
Kenya	-0.03	0.04	-0.10	0.04	-0.03	11916	-0.91	0.36
Mexico	-0.02	0.05	-0.12	0.09	-0.01	11916	-0.30	0.76
South Africa	-0.05	0.05	-0.14	0.05	-0.04	11916	-0.97	0.33
South Korea	-0.05	0.05	-0.14	0.04	-0.05	11916	-1.05	0.30
UAE	-0.03	0.04	-0.10	0.04	-0.03	11916	-0.84	0.40
UK	0.16	0.05	0.07	0.25	0.15	11916	3.51	< .001
US	0.12	0.04	0.03	0.20	0.11	11916	2.78	0.01

Simple effects of Wellbeing reason on Activism

Country	b	SE	Lower	Upper	β	df	t	p
Australia	-0.09	0.05	-0.18	0.00	-0.09	11916	-2.06	0.04
Brazil	0.02	0.05	-0.08	0.11	0.02	11916	0.34	0.73
China	-0.11	0.05	-0.20	-0.01	-0.10	11916	-2.22	0.03
India	0.02	0.03	-0.04	0.08	0.02	11916	0.66	0.51

Indonesia	-0.01	0.05	-0.09	0.08	0.00	11916	-0.12	0.91
Kenya	0.00	0.03	-0.06	0.06	0.00	11916	-0.03	0.97
Mexico	0.04	0.04	-0.05	0.12	0.04	11916	0.87	0.39
South Africa	0.01	0.04	-0.07	0.09	0.01	11916	0.24	0.81
South Korea	-0.02	0.05	-0.11	0.07	-0.02	11916	-0.47	0.64
UAE	0.01	0.03	-0.05	0.08	0.01	11916	0.39	0.70
UK	-0.10	0.05	-0.19	-0.01	-0.10	11916	-2.24	0.03
US	-0.12	0.04	-0.20	-0.04	-0.11	11916	-2.85	0.004

Table S22. Relative Importance Analyses in Each Country

Country	Predictor	RW	Lower	Upper	RS-RW (%)
Australia	Criterion = Consumer Environmental Behavior (R^2 = .23)				
	Intrinsic	0.0373	0.0222	0.0566	16.43
	Economic	0.0348	0.0205	0.0512	15.34
	Moral	0.0431	0.0276	0.0626	18.99
	Identity	0.0404	0.0258	0.0564	17.78
	Health	0.038	0.0249	0.0533	16.72
	Wellbeing	0.0335	0.0207	0.0494	14.74
	Criterion = Activism Environmental Behavior (R^2 = .14)				
	Intrinsic	0.0057	0.0028	0.0121	4.06
	Economic	0.0146	0.0072	0.0263	10.48
	Moral	0.0605	0.0396	0.0856	43.3
	Identity	0.0416	0.026	0.0613	29.76
	Health	0.0099	0.0054	0.0171	7.1
	Wellbeing	0.0074	0.0045	0.0116	5.29
Brazil	Criterion = Consumer Environmental Behavior (R^2 = .12)				
	Intrinsic	0.0025	0.0011	0.0057	2.11
	Economic	0.0041	0.0019	0.0075	3.44
	Moral	0.0339	0.0167	0.0576	28.6
	Identity	0.0589	0.0344	0.0916	49.71
	Health	0.0109	0.0038	0.0266	9.24
	Wellbeing	0.0082	0.0039	0.0181	6.9
	Criterion = Activism Environmental Behavior (R^2 = .06)				

	Intrinsic	0.0012	0.0003	0.0019	1.94
	Economic	0.0061	0.0019	0.0152	9.81
	Moral	0.0027	0.0009	0.01	4.31
	Identity	0.0387	0.0204	0.0645	62.12
	Health	0.0064	0.0025	0.0153	10.19
	Wellbeing	0.0073	0.0026	0.0164	11.63
China	Criterion = Consumer Environmental Behavior (R^2 = .23)				
	Intrinsic	0.028	0.0142	0.0451	12.19
	Economic	0.0379	0.0224	0.0567	16.5
	Moral	0.0526	0.0321	0.0855	22.92
	Identity	0.0308	0.018	0.0465	13.41
	Health	0.028	0.015	0.045	12.19
	Wellbeing	0.0522	0.0339	0.0738	22.78
	Criterion = Activism Environmental Behavior (R^2 = .07)				
	Intrinsic	0.0071	0.0019	0.0175	10.92
	Economic	0.003	0.0014	0.0069	4.59
	Moral	0.0468	0.0252	0.0743	71.53
	Identity	0.0026	0.0011	0.0057	3.99
	Health	0.0031	0.0011	0.0088	4.8
	Wellbeing	0.0027	0.0011	0.0061	4.16
India	Criterion = Consumer Environmental Behavior (R^2 = .24)				
	Intrinsic	0.0459	0.0344	0.0591	19.33
	Economic	0.0366	0.0271	0.0474	15.41
	Moral	0.0392	0.0299	0.0496	16.5
	Identity	0.041	0.0311	0.0521	17.29
	Health	0.0304	0.0223	0.0393	12.8
	Wellbeing	0.0443	0.0339	0.0564	18.66
	Criterion = Activism Environmental Behavior (R^2 = .07)				
	Intrinsic	0.0028	0.0015	0.0058	4.35
	Economic	0.0031	0.0019	0.0054	4.72
	Moral	0.0273	0.0181	0.0382	41.69
	Identity	0.0267	0.0177	0.0372	40.76
	Health	0.0028	0.0017	0.0047	4.26

	Wellbeing	0.0028	0.0017	0.0048	4.21
Indonesia		Criterion = Activism Environmental Behavior (R^2 = .07)			
	Intrinsic	0.0078	0.0018	0.0203	11
	Economic	0.0069	0.0023	0.0164	9.75
	Moral	0.028	0.0132	0.0483	39.51
	Identity	0.0055	0.0019	0.0133	7.76
	Health	0.0068	0.0023	0.0164	9.6
	Wellbeing	0.0158	0.0058	0.0308	22.37
		Criterion = Activism Environmental Behavior (R^2 = .04)			
	Intrinsic	0.0075	0.0012	0.0198	16.61
	Economic	0.0035	0.0012	0.0093	7.66
	Moral	0.0222	0.0095	0.0387	49.02
	Identity	0.0063	0.0015	0.0159	13.8
	Health	0.003	0.0011	0.0083	6.7
	Wellbeing	0.0028	0.0009	0.0097	6.21
Kenya		Criterion = Consumer Environmental Behavior (R^2 = .03)			
	Intrinsic	0.0034	0.0004	0.0139	9.91
	Economic	0.0047	0.0007	0.0165	13.65
	Moral	0.0024	0.0006	0.009	6.94
	Identity	0.0075	0.0011	0.021	21.68
	Health	0.0084	0.0017	0.0232	24.36
	Wellbeing	0.0081	0.0015	0.0209	23.45
		Criterion = Activism Environmental Behavior (R^2 = .003)			
	Intrinsic	0.0002	0	0.0007	5.34
	Economic	0.0002	0	0.0003	5.67
	Moral	0.0016	0.0001	0.0091	52.17
	Identity	0.0002	0	0.0006	6.67
	Health	0.0009	0.0001	0.006	27.67
	Wellbeing	0.0001	0	0.0001	2.48
Mexico		Criterion = Consumer Environmental Behavior (R^2 = .13)			
	Intrinsic	0.0062	0.0019	0.0169	4.85
	Economic	0.0207	0.0088	0.0388	16.28
	Moral	0.0374	0.0196	0.0608	29.51

	Identity	0.0375	0.0208	0.0607	29.53
	Health	0.0113	0.0051	0.0218	8.9
	Wellbeing	0.0139	0.0059	0.0267	10.94
	Criterion = Activism Environmental Behavior ($R^2 = .04$)				
	Intrinsic	0.0012	0.0004	0.0058	2.87
	Economic	0.0019	0.0007	0.0037	4.48
	Moral	0.0177	0.0065	0.0333	41
	Identity	0.0153	0.0054	0.0309	35.28
	Health	0.0022	0.0008	0.0051	5.2
	Wellbeing	0.0048	0.0014	0.0135	11.16
South Africa	Criterion = Consumer Environmental Behavior ($R^2 = .14$)				
	Intrinsic	0.0027	0.0012	0.009	1.93
	Economic	0.021	0.0094	0.0383	14.97
	Moral	0.056	0.0336	0.0836	39.95
	Identity	0.0393	0.0218	0.0613	28.04
	Health	0.0139	0.0065	0.028	9.91
	Wellbeing	0.0073	0.0037	0.0155	5.21
	Criterion = Activism Environmental Behavior ($R^2 = .08$)				
	Intrinsic	0.0029	0.0008	0.0113	3.49
	Economic	0.0152	0.0058	0.0296	18.33
	Moral	0.0325	0.0171	0.0532	39.27
	Identity	0.0207	0.0093	0.0377	25.02
	Health	0.0051	0.0024	0.0112	6.11
	Wellbeing	0.0064	0.0024	0.0151	7.78
South Korea	Criterion = Consumer Environmental Behavior ($R^2 = .23$)				
	Intrinsic	0.0233	0.0124	0.039	10.13
	Economic	0.0332	0.0187	0.0516	14.42
	Moral	0.0509	0.031	0.0744	22.11
	Identity	0.0435	0.0248	0.0655	18.91
	Health	0.0471	0.0289	0.0698	20.46
	Wellbeing	0.0322	0.0191	0.0481	13.98
	Criterion = Activism Environmental Behavior ($R^2 = .12$)				
	Intrinsic	0.0072	0.0029	0.0158	6.03

	Economic	0.0036	0.0018	0.0047	2.97
	Moral	0.0585	0.0357	0.0871	48.65
	Identity	0.0434	0.0233	0.0693	36.09
	Health	0.0035	0.0018	0.0048	2.94
	Wellbeing	0.004	0.0021	0.0056	3.32
UAE	Criterion = Consumer Environmental Behavior (R^2 = .24)				
	Intrinsic	0.0497	0.0294	0.0753	21.08
	Economic	0.0308	0.0176	0.0485	13.07
	Moral	0.0399	0.0246	0.0572	16.94
	Identity	0.0495	0.0306	0.0718	20.99
	Health	0.0246	0.014	0.039	10.43
	Wellbeing	0.0412	0.0247	0.0614	17.49
	Criterion = Activism Environmental Behavior (R^2 = .03)				
	Intrinsic	0.0024	0.0006	0.009	8.22
	Economic	0.0022	0.0007	0.0072	7.37
	Moral	0.0056	0.0012	0.0148	19
	Identity	0.0154	0.005	0.0316	51.81
	Health	0.0014	0.0003	0.0023	4.64
	Wellbeing	0.0027	0.0007	0.0091	8.97
UK	Criterion = Consumer Environmental Behavior (R^2 = .23)				
	Intrinsic	0.0209	0.0103	0.0367	8.94
	Economic	0.0304	0.0172	0.0479	13
	Moral	0.0407	0.0251	0.0599	17.41
	Identity	0.0512	0.031	0.0753	21.89
	Health	0.0562	0.0356	0.0781	24.05
	Wellbeing	0.0344	0.0207	0.0532	14.71
	Criterion = Activism Environmental Behavior (R^2 = .13)				
	Intrinsic	0.0055	0.0029	0.0116	4.13
	Economic	0.0183	0.0091	0.0319	13.73
	Moral	0.0468	0.0293	0.067	35.02
	Identity	0.028	0.015	0.044	20.93
	Health	0.0282	0.0151	0.045	21.09
	Wellbeing	0.0068	0.0042	0.0105	5.1

US					
	Criterion = Consumer Environmental Behavior (R^2 = .24)				
	Intrinsic	0.0053	0.0031	0.0099	2.27
	Economic	0.0465	0.03	0.0664	19.75
	Moral	0.0715	0.0499	0.0959	30.36
	Identity	0.0544	0.0362	0.0765	23.1
	Health	0.0357	0.0214	0.053	15.15
	Wellbeing	0.0221	0.0127	0.0351	9.37
	Criterion = Activism Environmental Behavior (R^2 = .15)				
	Intrinsic	0.004	0.002	0.0099	2.61
	Economic	0.0171	0.0082	0.0301	11.07
	Moral	0.0584	0.039	0.0808	37.85
	Identity	0.0403	0.0252	0.0582	26.16
	Health	0.0276	0.0156	0.043	17.92
	Wellbeing	0.0068	0.0043	0.0094	4.39

Note: RW is the raw relative weights for each motivation for valuing nature and can be interpreted as the proportion of variance in behavior that can be attributed to the corresponding motivation for valuing nature in the legend. Lower-CI and Upper-CI are the values for the 95% confidence interval for the estimate of the raw relative weight. RS-RW is a "rescaled" relative weight that represents the percentage of the model's R^2 that a given motivation accounts for.

Table S23. Main effects of country-level variable and their interactions with reasons for valuing nature in models predicting pro-environmental behavior

	Consumer Behavior						Activism Behavior					
Names	b	SE	Lower	Upper	t	p	b	SE	Lower	Upper	t	p
Environmental Performance	0.07	0.06	-0.05	0.19	1.10	.30	-0.13	0.08	-0.30	0.03	-1.57	.15
Intrinsic x Environmental Performance	-0.01	0.01	-0.03	0.00	-1.91	.06	-0.02	0.01	-0.04	0.00	-2.41	.02
Economic x Environmental Performance	0.00	0.01	-0.01	0.02	0.17	.86	0.03	0.01	0.01	0.05	2.54	.01
Moral x Environmental Performance	0.00	0.01	-0.01	0.02	0.28	.78	0.05	0.01	0.03	0.08	4.47	<.001
Identity x Environmental Performance	0.01	0.01	0.00	0.03	1.46	.15	0.05	0.01	0.03	0.07	4.18	<.001
Health x Environmental Performance	0.01	0.01	0.00	0.03	1.87	.06	0.04	0.01	0.02	0.06	3.44	< .01
Wellbeing x Environmental Performance	-0.01	0.01	-0.03	0.00	-1.36	.18	-0.03	0.01	-0.05	-0.01	-2.96	< .01
Pollution	0.05	0.06	-0.07	0.18	0.83	.42	0.09	0.09	-0.09	0.26	0.98	.35
Intrinsic x Pollution	0.03	0.01	0.02	0.04	4.64	< .001	0.02	0.01	0.00	0.03	1.53	.13
Economic x Pollution	0.00	0.01	-0.01	0.02	0.69	.49	-0.03	0.01	-0.05	-0.01	-2.97	< .01
Moral x Pollution	-0.01	0.01	-0.03	0.00	-1.48	.14	-0.04	0.01	-0.06	-0.02	-3.56	<.001
Identity x Pollution	-0.01	0.01	-0.03	0.00	-1.74	.08	-0.04	0.01	-0.07	-0.02	-4.23	<.001
Health x Pollution	-0.01	0.01	-0.02	0.01	-1.10	.27	-0.03	0.01	-0.05	-0.01	-2.82	< .01
Wellbeing x Pollution	0.02	0.01	0.01	0.04	3.03	< .01	0.02	0.01	0.00	0.04	1.79	.07
Life Expectancy	0.13	0.05	0.02	0.23	2.43	.04	-0.04	0.09	-0.22	0.14	-0.44	.67
Intrinsic x Life Expectancy	0.01	0.01	0.00	0.02	1.46	.15	-0.02	0.01	-0.04	0.00	-2.35	.02
Economic x Life Expectancy	0.01	0.01	-0.01	0.02	0.88	.38	0.01	0.01	-0.02	0.03	0.50	.62
Moral x Life Expectancy	0.01	0.01	-0.01	0.02	0.91	.36	0.06	0.01	0.04	0.08	4.98	< .001
Identity x Life Expectancy	0.01	0.01	-0.01	0.02	0.66	.51	0.05	0.01	0.03	0.07	4.17	< .001
Health x Life Expectancy	0.01	0.01	0.00	0.03	1.55	.12	0.02	0.01	0.00	0.04	1.82	.07
Wellbeing x Life Expectancy	0.01	0.01	-0.01	0.02	1.22	.22	-0.03	0.01	-0.05	-0.01	-2.69	.01
Economic Prosperity	0.05	0.06	-0.08	0.17	0.76	.46	-0.07	0.09	-0.25	0.11	-0.77	.46

Intrinsic x Economic Prosperity	0.01	0.01	0.00	0.02	1.25	.21	-0.02	0.01	-0.04	-0.00	-2.05	.04
Economic x Economic Prosperity	0.01	0.01	0.00	0.03	1.53	.13	0.01	0.01	-0.01	0.04	1.33	.18
Moral x Economic Prosperity	0.01	0.01	-0.01	0.02	1.04	.30	0.05	0.01	0.02	0.07	3.97	< .001
Identity x Economic Prosperity	0.01	0.01	-0.01	0.02	1.13	.26	0.03	0.01	0.01	0.05	2.81	.01
Health x Economic Prosperity	0.01	0.01	-0.01	0.02	0.87	.39	0.02	0.01	0.00	0.04	1.76	.08
Wellbeing x Economic Prosperity	0.00	0.01	-0.01	0.02	0.22	.83	-0.03	0.01	-0.05	0.00	-2.34	.02
Cultural Independence	0.02	0.07	-0.11	0.15	0.35	.74	-0.18	0.08	-0.33	-0.03	-2.32	.04
Intrinsic x Cultural Independence	-0.02	0.01	-0.03	0.00	-2.16	.03	-0.01	0.01	-0.03	0.01	-0.64	.52
Economic x Cultural Independence	0.01	0.01	0.00	0.03	1.67	.10	0.03	0.01	0.01	0.06	2.83	< .01
Moral x Cultural Independence	0.01	0.01	-0.01	0.03	1.22	.22	0.05	0.01	0.02	0.07	3.76	< .01
Identity x Cultural Independence	0.01	0.01	-0.01	0.03	0.94	.35	0.03	0.01	0.00	0.05	2.04	.04
Health x Cultural Independence	0.01	0.01	0.00	0.03	1.43	.15	0.04	0.01	0.02	0.06	3.14	< .01
Wellbeing x Cultural Independence	-0.02	0.01	-0.03	0.00	-2.10	.04	-0.03	0.01	-0.06	-0.01	-2.70	.01
Cultural Preference for Hierarchy	0.01	0.07	-0.12	0.14	0.10	.92	0.21	0.07	0.07	0.34	2.99	.01
Intrinsic x Cultural Preference for Hierarchy	0.03	0.01	0.02	0.05	4.46	< .001	0.02	0.01	0.00	0.04	1.62	.10
Economic x Cultural Preference for Hierarchy	-0.01	0.01	-0.03	0.01	-1.28	.20	-0.04	0.01	-0.06	-0.01	-3.10	< .01
Moral x Cultural Preference for Hierarchy	-0.02	0.01	-0.03	0.00	-1.79	.07	-0.06	0.01	-0.08	-0.04	-4.77	<.001
Identity x Cultural Preference for Hierarchy	-0.01	0.01	-0.02	0.01	-0.94	.35	-0.02	0.01	-0.05	0.00	-1.77	.08
Health x Cultural Preference for Hierarchy	-0.02	0.01	-0.04	-0.01	-2.63	.01	-0.04	0.01	-0.06	-0.02	-3.33	< .01
Wellbeing x Cultural Preference for Hierarchy	0.02	0.01	0.01	0.04	3.03	< .01	0.04	0.01	0.01	0.06	3.06	< .01

Note: These statistics come from 12 different models (6 country-level variables, which were always in entered in different models x 2 pro-environmental behaviors, which were always entered in different models).

Table S24. Complete Summary of Parameter Estimates for Mediating Variables in Path Analysis

Path			est	se	z	*p*	Lower	Upper
Identity	→ Awareness	→ Consumer	0.0213	0.0026	8.3	< .001	0.0167	0.0272
Moral	→ Awareness	→ Consumer	0.0160	0.0020	8.2	< .001	0.0124	0.0202
Economic	→ Awareness	→ Consumer	0.0085	0.0016	5.4	< .001	0.0056	0.0120
Wellbeing	→ Awareness	→ Consumer	0.0077	0.0015	5.2	< .001	0.0050	0.0109
Health	→ Awareness	→ Consumer	0.0068	0.0014	4.7	< .001	0.0042	0.0097
Intrinsic	→ Awareness	→ Consumer	0.0058	0.0012	4.7	< .001	0.0034	0.0082
Health	→ Concern	→ Consumer	0.0313	0.0031	10.2	< .001	0.0251	0.0378
Economic	→ Concern	→ Consumer	0.0280	0.0028	9.9	< .001	0.0227	0.0337
Identity	→ Concern	→ Consumer	0.0246	0.0026	9.4	< .001	0.0198	0.0302
Moral	→ Concern	→ Consumer	0.0215	0.0023	9.4	< .001	0.0169	0.0260
Wellbeing	→ Concern	→ Consumer	0.0186	0.0024	7.9	< .001	0.0144	0.0236
Intrinsic	→ Concern	→ Consumer	0.0110	0.0019	5.9	< .001	0.0076	0.0150
Identity	→ Self-efficacy	→ Consumer	0.0151	0.0018	8.4	< .001	0.0119	0.0191
Moral	→ Self-efficacy	→ Consumer	0.0077	0.0015	5.2	< .001	0.0049	0.0108
Health	→ Self-efficacy	→ Consumer	0.0054	0.0014	3.9	< .001	0.0028	0.0083
Economic	→ Self-efficacy	→ Consumer	0.0050	0.0014	3.4	< .001	0.0023	0.0080
Wellbeing	→ Self-efficacy	→ Consumer	0.0016	0.0013	1.2	0.23	-0.0010	0.0043
Intrinsic	→ Self-efficacy	→ Consumer	-0.0008	0.0012	-0.7	0.50	-0.0032	0.0015
Identity	→ Awareness	→ Activism	0.0335	0.0030	11.2	< .001	0.0278	0.0400
Moral	→ Awareness	→ Activism	0.0251	0.0024	10.6	< .001	0.0205	0.0300
Economic	→ Awareness	→ Activism	0.0134	0.0022	6.1	< .001	0.0093	0.0180
Wellbeing	→ Awareness	→ Activism	0.0121	0.0022	5.5	< .001	0.0081	0.0165
Health	→ Awareness	→ Activism	0.0106	0.0021	5.1	< .001	0.0068	0.0147
Intrinsic	→ Awareness	→ Activism	0.0091	0.0018	5.1	< .001	0.0055	0.0125
Health	→ Concern	→ Activism	0.0165	0.0024	7.0	< .001	0.0121	0.0216
Economic	→ Concern	→ Activism	0.0148	0.0021	6.9	< .001	0.0108	0.0192
Identity	→ Concern	→ Activism	0.0130	0.0019	6.7	< .001	0.0093	0.0170
Moral	→ Concern	→ Activism	0.0114	0.0016	6.9	< .001	0.0081	0.0146
Wellbeing	→ Concern	→ Activism	0.0098	0.0016	6.1	< .001	0.0069	0.0133
Intrinsic	→ Concern	→ Activism	0.0058	0.0011	5.3	< .001	0.0038	0.0080
Identity	→ Self-efficacy	→ Activism	0.0168	0.0019	8.7	< .001	0.0133	0.0207
Moral	→ Self-efficacy	→ Activism	0.0086	0.0016	5.4	< .001	0.0057	0.0119
Health	→ Self-efficacy	→ Activism	0.0060	0.0016	3.8	< .001	0.0031	0.0094
Economic	→ Self-efficacy	→ Activism	0.0055	0.0016	3.5	< .001	0.0026	0.0087
Wellbeing	→ Self-efficacy	→ Activism	0.0018	0.0015	1.2	0.23	-0.0012	0.0047
Intrinsic	→ Self-efficacy	→ Activism	-0.0009	0.0013	-0.7	0.51	-0.0036	0.0017

Contrast	est	SE	z	*p*	Lower	Upper
Moral→Awareness→Consumer VS. Economic→Awareness→Consumer	0.0092	0.0020	4.6	< .001	0.0056	0.0136
Moral→Self-efficacy→Consumer VS. Health→Self-efficacy→Consumer	0.0028	0.0021	1.3	0.19	-0.0011	0.0072
Moral→Awareness→Activism VS. Economic→Awareness→Activism	0.0117	0.0031	3.8	< .001	0.0057	0.0179
Moral→Self-efficacy→Activism VS. Health→Self-efficacy→Activism	0.0026	0.0022	1.2	0.25	-0.0019	0.0070

Note: Estimates of standardized indirect effects derived from path analysis. For the path analysis, we modeled a saturated model with the six motivations for valuing nature as predictors, three simultaneous mediators, and two outcome variables. All variables were standardized within the general population subsample before being entered into the model. Error calculation was made using 1000 bootstrap samples. "Est" represents the standardized path coefficient. "SS" represents standard error. The lower and Upper are the 95% confidence intervals for the standardized path coefficient.

Figure S1. Model of path analysis of how reasons for valuing nature affect pro-environmental behaviors via three mechanisms.

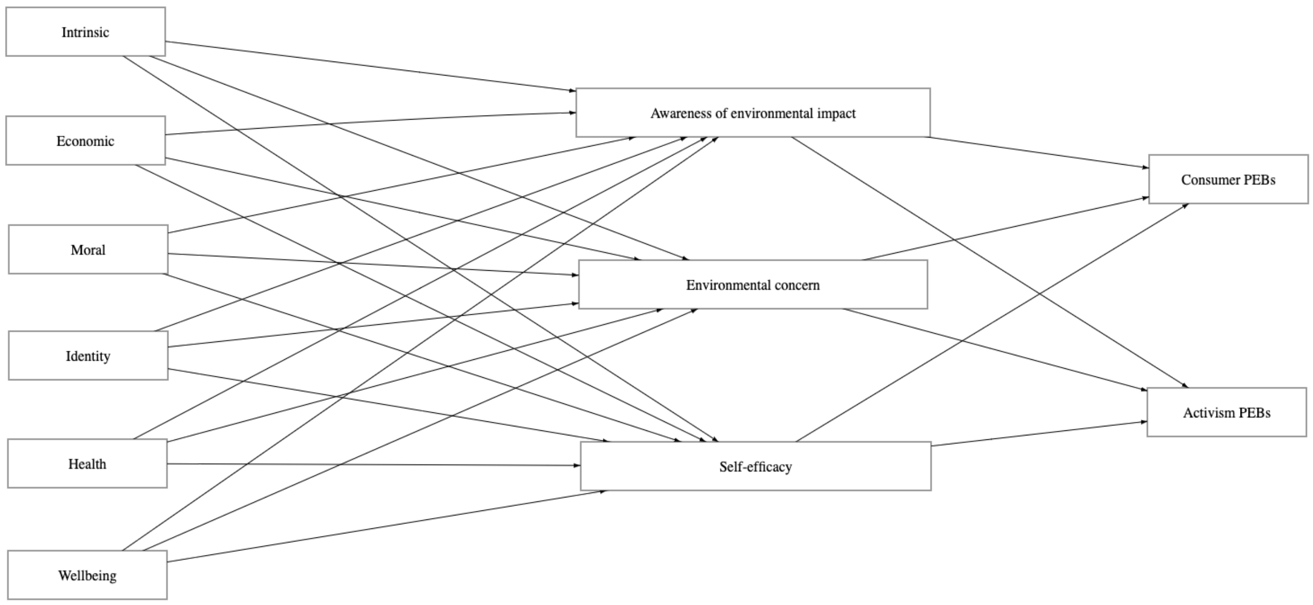

Note: This model shows the indirect pathways of the six reasons for valuing nature on the two pro-environmental behaviors, but not the direct effects of the reasons on the two behaviors (even though those direct effects were statistically modeled in the path analysis). We did not depict those direct effects in the image because we wanted the image to be as readable as possible.

Table S25. Exploratory factor analysis of the six reasons for valuing nature

	**Factor**			
	1	2	3	Uniqueness
Intrinsic	0.638			0.661
Economic			0.798	0.341
Moral				0.553
Identity		0.923		0.155
Health	0.558			0.452
Wellbeing	0.721			0.488

Note. 'Minimum residual' extraction method was used in combination with a 'oblimin' rotation. Factors were extracted based on parallel analysis

Table S26. Correlations between different reasons for valuing nature

	Intrinsic	Economic	Moral	Identity	Health	Wellbeing
Intrinsic	—	—	—	—	—	—
Economic	0.409	—	—	—	—	—
Moral	0.366	0.493	—	—	—	—
Identity	0.396	0.495	0.549	—	—	—
Health	0.426	0.558	0.481	0.525	—	—
Wellbeing	0.416	0.506	0.459	0.512	0.525	—

Note: All correlations significant at $p < .001$.

Table S27. Factor loadings for Consumer Behavior items

Item	Factor Loading	Uniqueness
Recycle	0.54	0.71
Avoid environmentally harmful products	0.58	0.67
Reusable bags	0.63	0.60
Non-car transportation	0.47	0.78

Note. 'Minimum residual' extraction method was used in combination with a 'oblimin' rotation. Model was fixed to one factor.

Table S28. Factor loadings for Activism items

Item	Factor Loading	Uniqueness
Share environmental issue on social media	0.87	0.25
Talk to family and friends	0.77	0.40
Donated money to environmental cause	0.48	0.77
Volunteered for environmental cause	0.56	0.69
Signed petition for environmental cause	0.42	0.82

Note. 'Minimum residual' extraction method was used in combination with a 'oblimin' rotation. Model was fixed to one factor. Because the items making up this scale are binary, the factor analysis is based on an underlying matrix of tetrachoric correlations, which is an imperfect but best practice for factor analysis with binary variables.
